# Deciphering Auditory Hyperexcitability in *Otogl* Mutant Mice Unravels an Auditory Neuropathy Mechanism

**DOI:** 10.1002/advs.202410776

**Published:** 2025-02-18

**Authors:** Mathilde Gagliardini, Sabrina Mechaussier, Carolina Campos Pina, Monica Morais, Olivier Postal, Philippe Jean, Typhaine Dupont, Amrit Singh‐Estivalet, Shéhanie Udugampolage, Cyril Scandola, Elisabeth Verpy, Baptiste Libé‐Philippot, Talya C. Inbar, Joanna Schwenkgrub, Carla Maria Barbosa Spinola, Raphaël Etournay, Aziz El‐Amraoui, Brice Bathellier, Adeline Mallet, Sedigheh Delmaghani, Fabrice Giraudet, Christine Petit, Boris Gourévitch, Paul Avan, Nicolas Michalski

**Affiliations:** ^1^ Université Paris Cité, Institut Pasteur, AP‐HP, INSERM, CNRS, Fondation Pour l'Audition, Institut de l'Audition, IHU reConnect Plasticity of Central Auditory Circuits Paris F‐75012 France; ^2^ Sorbonne Université Collège Doctoral Paris F‐75005 France; ^3^ Université Paris Cité Institut Pasteur, AP‐HP, INSERM, CNRS, Fondation Pour l'Audition, Institut de l'Audition, IHU reConnect, Auditory Therapies Innovation Laboratory Paris F‐75012 France; ^4^ Institut Pasteur Université Paris Cité Ultrastructural Bioimaging Unit Paris F‐75015 France; ^5^ Institut Pasteur IUF, Université Paris Cité, Human Genetics and Cognitive Functions Paris F‐75015 France; ^6^ Université Paris Cité, Institut Pasteur, AP‐HP, INSERM, CNRS, Fondation Pour l'Audition, Institut de l'Audition, IHU reConnect Auditory System Dynamics and Multisensory Processing Paris F‐75012 France; ^7^ Université Paris Cité, Institut Pasteur, AP‐HP, INSERM, CNRS, Fondation Pour l'Audition, Institut de l'Audition, IHU reConnect Cochlear Development and Therapeutic Perspectives Paris F‐75012 France; ^8^ Université Paris Cité, Institut Pasteur, AP‐HP, INSERM, CNRS, Fondation Pour l'Audition, Institut de l'Audition, IHU reConnect, Progressive Sensory Disorders Pathophysiology and Therapy Paris F‐75012 France; ^9^ Laboratoire de Biophysique Neurosensorielle, INSERM 1107 Université Clermont Auvergne Clermont‐Ferrand F‐63000 France; ^10^ Service de Génétique Médicale CHU de Clermont‐Ferrand Clermont‐Ferrand F‐63000 France; ^11^ Collège de France Paris F‐75005 France; ^12^ Université Paris Cité, Institut Pasteur, AP‐HP, INSERM, CNRS, Fondation Pour l'Audition, Institut de l'Audition, IHU reConnect Centre de Recherche et d'Innovation en Audiologie Humaine Paris F‐75015 France

**Keywords:** auditory neuropathy, low spontaneous rate spiral ganglion neurons, middle ear muscle reflex, Otogelin‐like, reemerging auditory brainstem responses

## Abstract

Auditory neuropathies affect the spiral ganglion neurons of the auditory nerve or their synapses with the sensory hair cells, distorting the sound information transmitted from the ear to the brain. Deciphering the underlying pathophysiological mechanisms remains challenging owing to the diversity of spiral ganglion neuron subtypes and associated central auditory circuits. An auditory neuropathy mechanism is unraveled by investigating the origin of auditory hyperexcitability in a mouse model for hereditary congenital deafness. *Otogl* encodes the large Otogelin‐like protein, which is related to secreted epithelial mucins and is implicated in the mechanical stimulation of cochlear outer hair cells. Heterozygous *Otogl* ^+/−^ mutant mice display auditory hyperexcitability, highlighted by their susceptibility to audiogenic seizures induced by loud sounds. It is shown that *Otogl* is transiently expressed in a subpopulation of spiral ganglion neurons during cochlear development. Despite their apparently normal hearing, *Otogl^+/−^* mice display poor activation of the spiral ganglion neurons processing loud sounds and an elevation of the activation threshold of the middle the ear muscle reflex that attenuates loud sounds. The findings reveal how a neuropathy affecting spiral ganglion neurons specialized in loud sound processing and associated with the middle the ear muscle reflex can manifest itself as auditory hyperexcitability.

## Graphical abstract

By investigating auditory hyperexcitability in a mouse model for hereditary deafness, this study identified a subpopulation of afferent neurons of the auditory nerve marked by *Otogl* expression. Despite their apparently normal hearing, *Otogl* ^+/−^ mice display poor activation of afferent neurons processing loud sounds and an elevation of the middle the ear muscle reflex activation threshold, identifying a mechanism of auditory neuropathy.

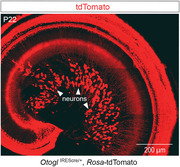

## 1 Introduction

Auditory neuropathies form a large spectrum of congenital and acquired disorders, mostly affecting the spiral ganglion neurons (SGNs) of the auditory nerve and/or the synapses they form with the auditory sensory hair cells. These disorders distort the sound information transmitted from the cochlea to the brain. Affected patients have hearing dysfunctions characterized by major difficulties in understanding speech not accounted by auditory thresholds.^[^ ^1^, ^2^, ^3^, ^4^ ^]^ The diversity of their aetiologies and clinical features constitutes a major challenge in patient stratification and efforts to decipher the underlying pathophysiological mechanisms.

There are two types of SGNs: type I (90%–95% of SGNs), which contact inner hair cells (IHCs), a single row of genuine sensory cells, and type II (5%–10% of SGNs), which contact the outer hair cells (OHCs) organized into three rows and involved in cochlear amplification. These myelinated neurons carry encoded information about the spectral, temporal and intensity characteristics of sound to the central auditory system. They have been classified into three functional categories based on activation thresholds and the spontaneous rate (SR) of action potential firing: high, medium, and low SR neurons, with low, medium, and high sound intensity activation thresholds, respectively.^[^ ^5^, ^6^ ^]^ The basolateral region of IHCs contains 10 to 30 synaptic active zones, each facing the single dendritic bouton of a type I neuron. All IHCs are in contact with the three types of neurons, resulting in coverage of the entire dynamic range of sound intensity coding.^[^ ^7^ ^]^ Single‐cell transcriptomic studies in the murine cochlea have confirmed the subdivision of type I SGNs into three populations with different transcriptomic signatures; types IA, IB, and IC.^[^ ^8^, ^9^, ^10^ ^]^ At the apical and middle parts, there are ≈30% type IA, 45% type IB, and 25% type IC neurons; while at the base of the cochlea, there are more type IA neurons (45%), fewer type IB neurons (25%) but a similar proportion of type IC neurons (30%).^[^ ^9^ ^]^ While type IC neurons defined by “Lypd1” expression have been shown to have functional characteristics of low SR neurons, the degree of correlation between the molecularly defined type IA and IB neurons and the functionally defined high and middle SR neurons, respectively, needs further investigation.^[^ ^11^ ^]^ Moreover, the lack of early molecular markers specific for each type I neuron subtype makes it difficult to determine the contributions of spontaneous activity before hearing onset and sound‐evoked activity after hearing onset to the refinement and maturation of the type I neurons.^[^ ^11^, ^12^, ^13^, ^14^ ^]^ Finally, we know little about the connectivity patterns of type I neurons in the cochlear nucleus (CN),^[^ ^15^ ^]^ the obligatory first relay station of the central auditory system. Therefore, it cannot be ruled out that each type I neuron subtype presents diverse projections, adding subdivisions to their current classification.

High‐threshold low‐SR neurons are thought to play the most crucial role in hearing in noisy environments, whereas low‐threshold high‐SR neurons have a saturated firing rate in these conditions, limiting the sound information they can carry.^[^ ^16^ ^]^ Moreover, low SR neurons decrease in number more rapidly than high SR neurons with aging in gerbils.^[^ ^17^ ^]^ Noise overexposure in rodents has also been shown to lead to presynaptic auditory neuropathy without hearing threshold alteration, due to the selective loss of synaptic connections between low SR neurons and IHCs.^[^ ^18^, ^19^ ^]^ This preclinical model for so‐called ‘hidden hearing loss’^[^ ^20^ ^]^ — auditory neuropathy with normal auditory thresholds and normal auditory brainstem responses (ABRs) at low sound levels — clearly implicates low SR SGNs in this condition.^[^ ^18^, ^19^ ^]^ However, hidden hearing loss is poorly reproducible between species and difficult to relate to the noise exposure history of humans.^[^ ^21^ ^]^ Furthermore, this model fails to address two issues: the mechanisms of postsynaptic neuropathies in general and the identity of the central auditory circuits activated downstream from low SR neurons and their associated functions.

These issues are of prime importance in humans, as difficulty understanding speech in noisy environments is the first auditory defect observed in age‐related hearing loss,^[^ ^22^ ^]^ a typical context for hidden auditory neuropathy. However, human aging cannot provide as clean a model like a specific disorder of low SR neurons, as progressive hearing threshold elevations rapidly blur the functional pattern. A complete understanding of the perceptual consequences of low SR neuron deficits would require the deciphering of the neuronal circuits malfunctioning in auditory neuropathy. Nonetheless, some physiological and perceptual consequences for auditory excitability can be inferred from the range of intensities to which low SR neurons respond. Deficits of low SR neurons should impair the processing of loud noise. They may also affect the middle‐ear muscle reflex (MEMR) triggered by loud noise and thought to provide the ear protection^[^ ^23^, ^24^ ^]^ and denoising.^[^ ^25^ ^]^ Further evidence of links between low SR neurons and the neural circuits controlling auditory excitability is provided by hyperacusis, a low tolerance of moderate sound levels, possibly associated with noise‐related hidden hearing loss.^[^ ^26^ ^]^

The development of a genetic model of auditory neuropathy would circumvent this translational issue, as the mouse cochlea often accurately reproduces the pathophysiology of human genetic forms of deafness.^[^ ^27^, ^28^ ^]^ We screened mutant mouse lines for susceptibility to audiogenic seizures — reflex seizures induced by loud sounds^[^ ^29^, ^30^ ^]^ — indicative of an abnormal control of auditory excitability. We found that mice carrying mutations of the gene encoding otogelin‐like, a large protein related to secreted epithelial mucins implicated in hair bundle top‐connectors and the crown attaching OHCs to the tectorial membrane,^[^ ^31^, ^32^ ^]^ were highly susceptible to audiogenic seizures. Both homozygous *Otogl* ^−/−^ mutant mice with moderate to severe hearing loss and heterozygous *Otogl* ^+/−^ mutant mice with normal hearing thresholds^[^ ^31^ ^]^ were susceptible to audiogenic seizures. *Otogl* ^+/−^ mutant mice are, thus, an attractive model for investigating auditory excitability mechanisms. We found that inactivation of *Otogl*, an early marker of a principally type IC population of SGNs, impaired high‐threshold low SR neuron activation, identifying a new mechanism underlying auditory neuropathy.

## 2 Results

### 2.1 *Otogl* Mutant Mice are Highly Susceptible to Audiogenic Seizures

**Figure 1:**
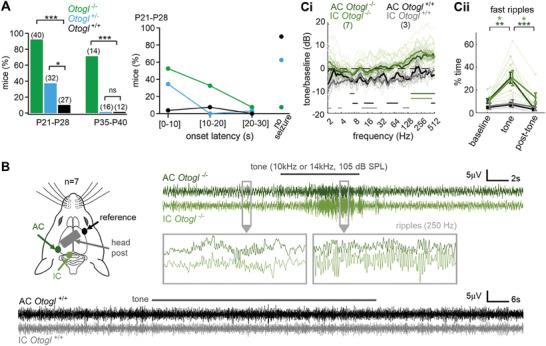
Audiogenic seizures in *Otogl* mutant mice. A, left) Proportion of *Otogl* ^+/+^, *Otogl* ^+/−^, and *Otogl* ^−/−^ mice displaying susceptibility to audiogenic seizures on P21–P28 and P35–P42. A, right) Distribution of onset latency for audiogenic seizures in *Otogl* ^+/+^, *Otogl* ^+/−^, and *Otogl* ^−/−^ mice on P21–P28. B, left) For electroencephalography (EEG) recordings, pins were implanted chronically at the surface of the auditory cortex (AC) and the inferior colliculus (IC) in head‐fixed awake animals. B, right) Individual examples of the electroencephalography (EEG) signal (sampling rate 5 kHz) obtained during an audiogenic seizure in an *Otogl* ^−/−^ mouse and during a tone presentation in an *Otogl* ^+/+^ mouse. In silence, several isolated spontaneous fast ripples occurred with oscillations ≈250 Hz, in both the auditory cortex and inferior colliculus of *Otogl* ^−/−^mice (bottom left box). When a tone at 10 or 14 kHz (depending on the animal) was presented at 105 dB SPL, an audiogenic seizure was induced despite the severe deafness of *Otogl* ^−/−^ mutant mice, and this seizure was characterized by an increase in the amplitude and duration of the ripples on EEG signal in both the auditory cortex and inferior colliculus (right box). The tone was stopped as soon as behavioural signs of the crisis became visible on the video recording of the animals. Ci) Graph showing the ratio of the spectrum during tone presentation to that in the 20 s before the tone in *Otogl* ^+/+^ and *Otogl* ^−/−^ mice. In both areas, the increase in energy concerned principally the 150–500 Hz range. Frequency domains of significant difference with the 0 dB horizontal dashed line (*p* < 0.05) are indicated by horizontal bars. Cii) Percentage duration of ripples in *Otogl* ^+/+^ and *Otogl* ^−/−^ mice before, during and after tone presentation. Ripples typically occupied 10% of the time in both brain areas, increasing to 30% of the time, on average, during tone presentation in *Otogl* ^−/−^ mutant mice. Numbers in brackets indicate the number of animals tested (A,C). AC, auditory cortex; EEG, electroencephalography; IC, inferior colliculus; ns, non‐significant.

We screened mutant mouse lines carrying pathogenic variants of deafness genes for susceptibility to audiogenic seizures. *Otogl* ^−/−^ and *Otogl* ^+/−^ mutant mice were highly susceptible to audiogenic seizures elicited by 10–11 kHz pure tones at ≈100 dB between postnatal days 21 and 28 (P21 and P28), a period of enhanced plasticity in mice.^[^ ^33^ ^]^ *Otogl* ^+/−^ mice have normal auditory thresholds, whereas *Otogl* ^−/−^ mice display an auditory threshold elevation by 50–60 dB due to OHC dysfunction.^[^ ^31^ ^]^ Audiogenic seizures were observed in almost all *Otogl* ^−/−^ mice (92.5%, *n* = 40). *Otogl* ^+/−^ mice were also significantly more susceptible to audiogenic seizures (37.5%, *n* = 32) than wild‐type *Otogl* ^+/+^ littermates (11.1%, *n* = 27, Fisher's exact test, *p* = 0.034) (**Figure** **1**A). Despite their hearing loss, *Otogl* ^−/−‐^ mutant mice were so susceptible that seizures began within 10 s of sound stimulus presentation in 52% of these mice (Figure 1A). *Otogl* ^+/−^ mice ceased to be susceptible to audiogenic seizures by P35–P40 (0%, *n* = 16), when the central auditory pathways reached full maturity,^[^ ^34^, ^35^ ^]^ but *Otogl* ^−/−^ mice remained susceptible to audiogenic seizures at this age (71%, *n* = 14, Fisher's exact test *p* = 0.0002) (Figure 1A). These findings indicate that the auditory system of *Otogl* ^−/−^ and *Otogl* ^+/−^ mice is hypersensitive to sound.

We then characterized the electrophysiological signature of audiogenic seizures in *Otogl* ^−/−^ mice by placing epidural electrode pins at the surface of the auditory cortex and the inferior colliculus of awake animals (Figure 1B). In quiet conditions, sparse, fast, and short oscillatory ripples resembling interictal events were observed at ≈250 Hz in both areas of *Otogl* ^−/−^ mice (*p* < 0.05, Student's *t*‐test; Figure 1B,Ci). Presentation of a loud tone to induce an audiogenic seizure increased the amplitude and duration of these ripples (Figure 1B,Cii) in *Otogl* ^−/−^ mice (*p* = 0.0024 in the auditory cortex and *p* = 0.0185 in the inferior colliculus, Student's *t*‐test with Welch's correction). The electroencephalography (EEG) spectrum changed during seizures, with a significant decrease of energy in the theta band (7‐8 Hz) in the auditory cortex of *Otogl* ^−/−^ mice and a significant increase in the 150–500 Hz range, corresponding to fast ripples, in both the inferior colliculus and auditory cortex of *Otogl* ^−/−^ mice (Student's *t*‐test; Figure 1Ci). In *Otogl* ^+/+^ mice, tone presentation did not lead to a ripple increase (Student's *t*‐test, *p* > 0.09) and provoked a decrease of EEG power in many frequency bands suggesting the recruitment of auditory inhibitory neuronal circuits (Figure 1B,C).

### 2.2 *Otogl* is Expressed in Spiral Ganglion Neurons

**Figure 2:**
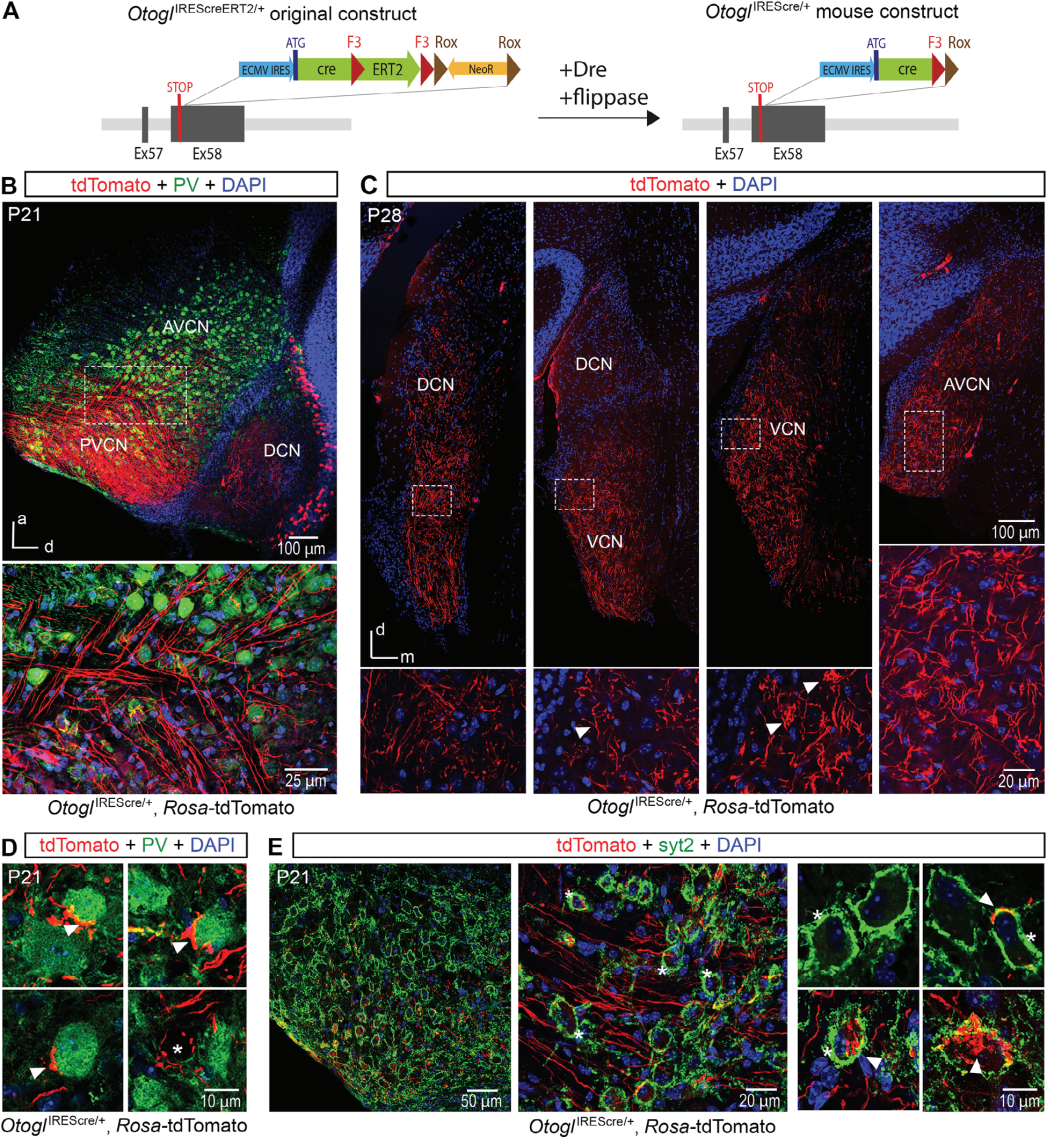
*Otogl* is a marker of a population of SGNs within the cochlear nucleus. A) Diagram of the original *Otogl*‐IREScreERT2 genetic construct and the generation of *Otogl*‐IREScre through successive Dre and flippase DNA recombinations. *Otogl* ^IREScre/+^:*Rosa‐*tdTomato mice were generated by crossing *Otogl* ^IREScre/+^ mice with *Rosa*‐tdTomato mice expressing the red fluorescent protein tdTomato conditionally for fate mapping. B) Sagittal section of the cochlear nucleus (CN) of a P21 *Otogl* ^IREScre/+^:*Rosa‐*tdTomato mouse immunostained for PV (green) (top), with a detailed view of the inset showing the bifurcation of the SGNs between the ascending and descending branches. C) Coronal section of the CN of a P28 *Otogl* ^IREScre/^:*Rosa‐*tdTomato mouse along the posterior (left) to anterior (right) axis (top), with expanded views of the insets showing details of tdTomato‐positive neurons (bottom). Arrowheads indicate the presence of a series (left) and small bouquets (right) of tdTomato‐positive synaptic boutons. D) Enlarged view of the principal neurons of the CN from a P21 *Otogl* ^IREScre/+^:*Rosa‐*tdTomato mouse immunostained for PV (green). Arrowheads indicate the presence of tdTomato‐positive synaptic boutons around the principal neurons of the CN. * indicates one of the principal neurons of the CN that is negative for PV and contacted by several tdTomato‐positive synaptic boutons. E) Coronal sections of the CN from a P21 *Otogl* ^IREScre/+^:*Rosa‐*tdTomato mouse immunostained for the endbulb of Held synaptic marker synaptotagmin‐2 (green). Some endbulbs of Held are pinpointed by asterisks (middle and right panels). Arrowheads indicate tdTomato‐positive synaptic connections (right panel), which contact postsynaptic bushy cells but do not overlap with the endbulbs of Held. Cell nuclei are stained in blue (DAPI). a, anterior; AVCN, anteroventral cochlear nucleus; d, dorsal; DCN, dorsal cochlear nucleus; m, middle; PVCN, posteroventral cochlear nucleus; VCN, ventral cochlear nucleus.

We investigated the anatomical regions underlying sound hypersensitivity in *Otogl* ^+/−^ and *Otogl* ^−/−^ mutant mice by generating a knock‐in mouse line expressing the cre recombinase under the control of the *Otogl* promoter. As monoallelic *Otogl* inactivation induces an audiogenic seizure phenotype, an IRES‐cre construct was knocked in at the 3’ end of the gene, for co‐expression of the endogenous *Otogl* and *cre* under the control of the same *Otogl* promoter (*Otogl* ^IREScre/+^ mice) (**Figure** **2**A). We crossed the *Otogl* ^IREScre/+^ mice with *Rosa*‐tdTomato Ai9 mice,^[^ ^36^ ^]^ which express the red fluorescent tdTomato protein upon cre‐driven recombination, for fate mapping. In these mice, tdTomato expression will start upon cre activation and will remain active even if *Otogl* expression ceases. Surprisingly, cells expressing tdTomato were sparsely distributed across the brains of P12–P40 *Otogl* ^IREScre/+^:*Rosa‐*tdTomato mice (Figure S1, Supporting Information), in a small fraction of olfactory bulb granular cells (Figure S2A, Supporting Information), and some peristriatal area neurons without projections onto the striatum (Figure S2B, Supporting Information). Given the severity and rapid onset of the audiogenic seizures, the small number of cells labeled in the brain led us to explore the auditory pathways in greater detail.

No tdTomato‐positive cells were detected within the central auditory system including the auditory cortex, thalamus, inferior colliculus, lateral lemniscus, or superior olivary complex of *Otogl* ^IREScre/+^:*Rosa‐*tdTomato mice (Figure S1and S3, Supporting Information). However, many axons in the CN were tdTomato‐positive, indicating *Otogl* promoter activity in some SGNs of the auditory nerve (Figure 2B,C). Analyses of coronal or sagittal slices from *Otogl* ^IREScre/+^:*Rosa‐*tdTomato mice showed that these axons were predominantly located in the ventral CN and less numerous in the dorsal part (Figure 2B).

We then investigated the shape of the synaptic terminals of these neurons. Many putative synaptic connections were detected on the dorsal border of the ventral CN. They appeared as individual small boutons, occasionally organized into series or small bouquets (Figure 2C). We identified relatively large boutons positive for both tdTomato and parvalbumin (PV), a marker of SGNs and ventral CN bushy cells (Figure 2D). However, with the presynaptic Ca^2+^ sensor synaptotagmin‐2 (syt2), a marker of the giant synaptic endbulbs of Held contacting bushy cells,^[^ ^9^, ^37^ ^]^ no double‐labeling was observed with tdTomato either on P21 at the age of maximal sensitivity of *Otogl* ^+/−^ mutant mice to audiogenic seizures (0%, *n* = 207 counted endbulbs of Held; Figure 2E) or on P35 when the CN is fully mature (0%, *n* = 231 counted endbulbs of Held; Figure S4, Supporting Information). Notably, some tdTomato‐positive neurons formed syt2‐positive small boutons on CN bushy cells within or next to the large endbulb of Held (Figure 2E).

**Figure 3:**
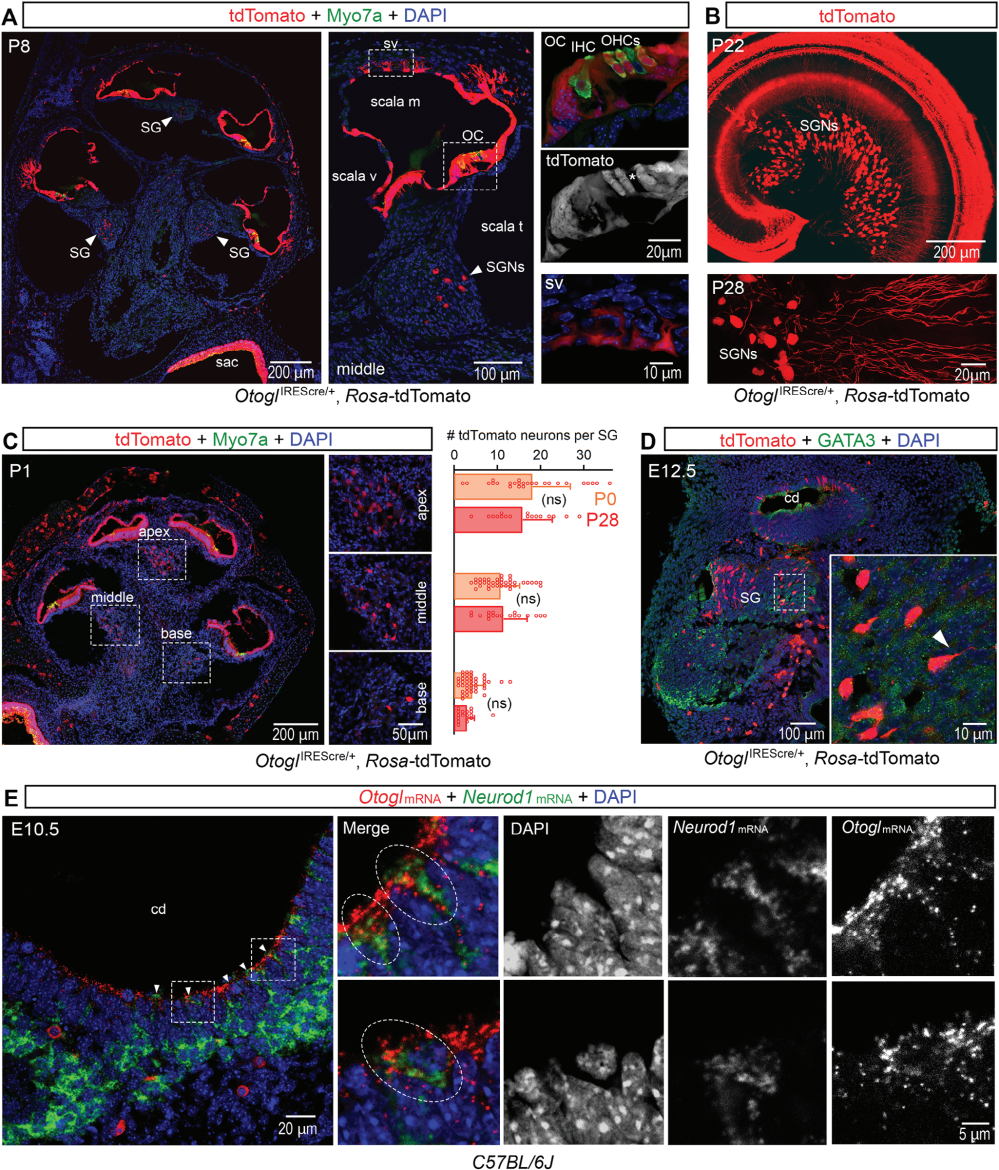
*Otogl* is an early marker of a population of SGNs. A) Longitudinal cross‐section of the cochlea of a P8 *Otogl* ^IREScre/+^:*Rosa‐*tdTomato mouse immunostained for Myosin7a (Myo7a) (green) (left) with an enlarged view of a cochlear canal (middle) and expanded views of the insets (right) showing details of tdTomato‐positive cells in the neurosensory epithelium, the organ of Corti (top right), and stria vascularis (bottom right). Arrowheads point to the spiral ganglia. * indicates an OHC with no tdTomato expression in the middle cochlear canal (middle right). B) Z‐projection of a cochlear whole mount from a P22 *Otogl* ^IREScre/+^:*Rosa‐*tdTomato mouse (top) and acquisition of SGNs from a P28 *Otogl* ^IREScre/+^:*Rosa‐*tdTomato mouse (bottom). C, left) Longitudinal cross‐section of the cochlea of a P1 *Otogl* ^IREScre/+^:*Rosa‐*tdTomato mouse immunostained for Myosin7a (green) with an enlarged view of the apical, middle, and basal spiral ganglia. C, right) Bar graph comparing the number of tdTomato neurons per spiral ganglion between P0, when the cochlea has almost reached its final length but spontaneous and auditory‐elicited activity has not yet begun,^[^ ^40^, ^41^ ^]^ and P28, when the cochlea is mature in the apex, middle, and base of the cochlea in *Otogl* ^IREScre/+^:*Rosa‐*tdTomato mice. D) Longitudinal cross‐section of the inner the ear of an E12.5 *Otogl* ^IREScre/+^:*Rosa‐*tdTomato mouse embryo immunostained for GATA3 (green) with an enlarged view of neurons from the cochleo‐vestibular ganglion. The arrowhead points to a neuron with its axon. E, left) Coronal cross‐section of the otic vesicle of an E10.5 mouse embryo stained for *Otogl* (red) and Neurod1 (green) mRNA. E, right) Two highlighted regions with cells co‐expressing *Otogl* and Neurod1 are indicated by dashed ellipses. Cell nuclei are stained in blue (DAPI). cd, cochlear duct; IHC, inner hair cell; OC, organ of Corti; OHCs, outer hair cells; ov, otic vesicle; sac, saccule; scala m, scala media; scala t, scala tympani; scala v, scala vestibuli; SG, spiral ganglion; SGNs, spiral ganglion neurons; sv, stria vascularis; ns, non‐significant.

We then searched for the cell bodies of the SGNs expressing tdTomato in the spiral ganglia of *Otogl* ^IREScre/+^:*Rosa‐*tdTomato mice (**Figure** **3**; Figure S5, Supporting Information). The *Otogl* promoter was active in many cochlear cell types during the course of their development, consistent with the single‐cell transcriptomic data.^[^ ^38^ ^]^ In line with the known functions of otogelin‐like in the cochlea,^[^ ^31^, ^32^ ^]^ tdTomato was expressed in several cell types of the sensory epithelium involved in tectorial membrane formation and maintenance, including interdental cells, supporting cells, and hair cells (Figure 3A; Figure S5,S6, Supporting Information). No tdTomato was detected in the IHCs or OHCs of the basal region of the cochlea or in some OHCs of the middle region (Figure 3A, Figure S6, Supporting Information). The *Otogl* promoter was also active in the root cells and some marginal cells of the stria vascularis (Figure S5 and S7, Supporting Information). Large cell bodies expressing tdTomato were found in the spiral ganglion along the entire tonotopic axis (Figure 3B‐C). In *Otogl* ^IREScre/+^:*Rosa‐*tdTomato mice, the number of tdTomato‐positive neurons in the apex, middle, and base of the cochlea did not differ between P0–P1 and P28 (*p* = 0.33, *p* = 0.66, and *p* = 0.07, respectively, Student's *t‐*test with Welch's correction), suggesting that *Otogl* was an early marker of this neuronal population (Figure 3C). We therefore investigated the age at which tdTomato expression began during embryonic development. No tdTomato‐positive cells were detected in the otic vesicles of E10.5 *Otogl* ^IREScre/+^:*Rosa‐*tdTomato embryos (Figure S8A, Supporting Information). However, some tdTomato‐positive cells were detected in the cochlear duct on E11.5 and by E12.5, some neurons of the cochlear ganglion, the future spiral ganglion, were tdTomato‐positive (Figure 3D; Figure S8A, Supporting Information). We then analyzed *Otogl* mRNA levels by in situ RNA hybridization assays (RNAscope) to determine whether *Otogl* expression was turned off early in SGN development (Figure S8B, Supporting Information). *Otogl* mRNA was detected in the cochlear duct and/or sensory epithelium from E10.5 to postnatal ages, but not in the developing SGNs (from E10.5 to P20) (Figure S8B, Supporting Information). However, cells co‐expressing *Otogl* and Neurod1 mRNA, a marker of delaminating neuroblasts from the otic vesicle,^[^ ^39^ ^]^ were found in E10.5 otic vesicles (Figure 3E), suggesting that neuroblasts transiently express *Otogl* early on, before their delamination and migration.

### 2.3 *Otogl*‐Expressing Spiral Ganglion Neurons are Principally Type IC

**Figure 4:**
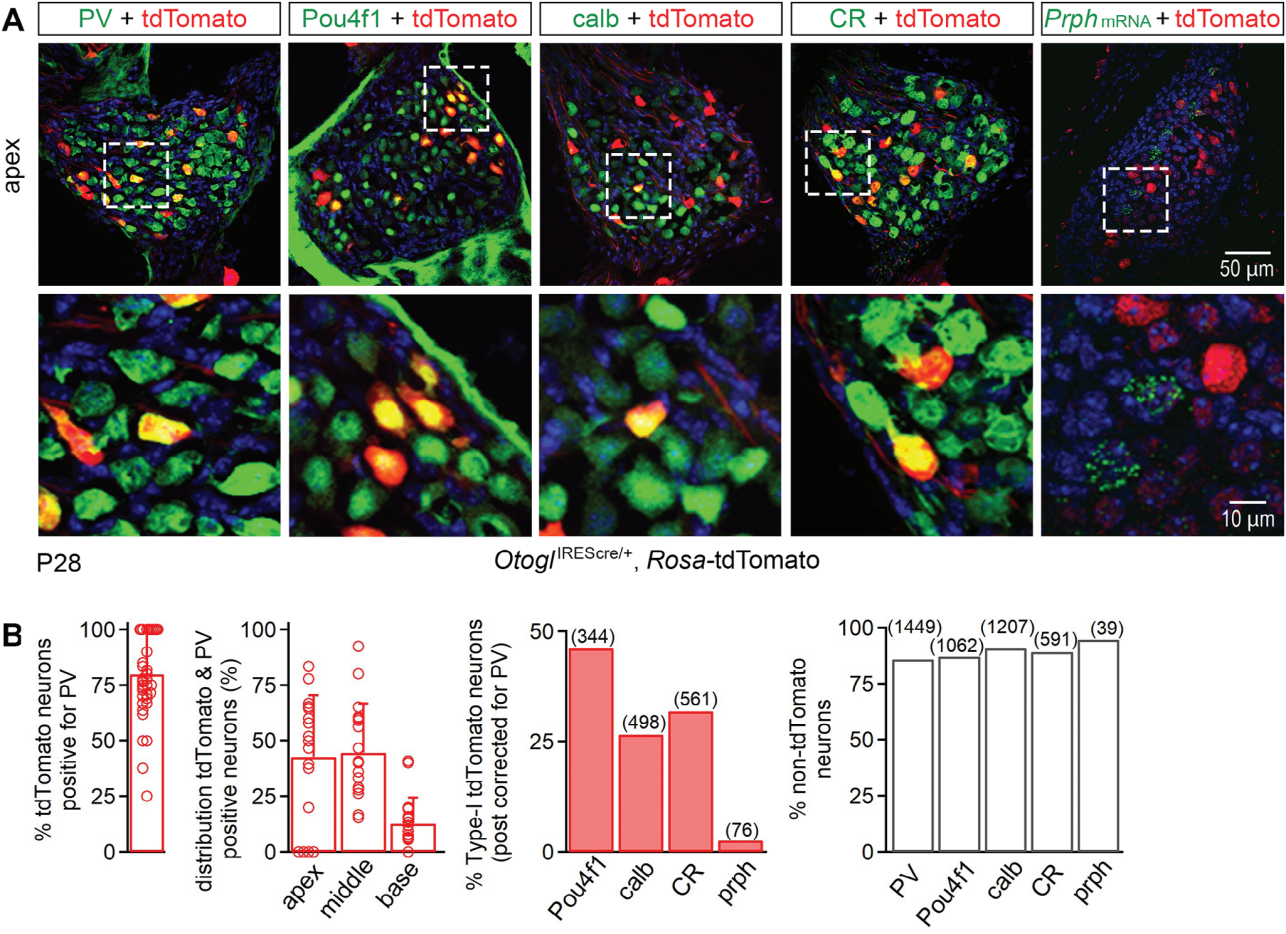
Molecular characterization of tdTomato‐positive neurons in *Otogl* ^IREScre/+^:*Rosa‐*tdTomato mice. A) Z‐projections of the apical cochlear spiral ganglia of a P28 *Otogl* ^IREScre/+^:*Rosa‐*tdTomato mouse immunostained for PV, Pou4f1, calb, or CR, or stained for *Prph* mRNA (top) with enlarged views of the insets showing details of SGNs (bottom). B) Bar graphs showing the percentage of tdTomato‐positive neurons positive for PV, the distribution of PV neurons positive for tdTomato at the apex, middle, and base of the cochlea, the percentage of tdTomato‐positive neurons also positive for Pou4f1, calb, CR, or *Prph* mRNA, and the percentage of tdTomato‐negative neurons positive for Pou4f1, calb, CR, or *Prph* mRNA. Cell nuclei are stained in blue (DAPI). Numbers in brackets indicate the number of neurons counted.

We then investigated the SGN subtypes in which the *Otogl* promoter was activated, by staining cochlear sections from *Otogl* ^IREScre/+^:*Rosa‐*tdTomato mice with markers displaying little overlap between the major defined subtypes on P28: peripherin encoded by *Prph* (type II), calbindin (calb) encoded by *Calb1* (type IB), calretinin (CR) encoded by *Calb2* (type IA), and Pou4f1 (type IC).^[^ ^9^, ^10^, ^42^ ^]^ About 11.5% of the cell bodies positive for PV, a marker of type I and II neurons, expressed tdTomato (*n* = 7094 cells counted) (**Figure** **4**A,B). There were more tdTomato‐positive neurons at the apex (42.7 ± 27.6%, *n* = 18 spiral ganglia) and middle of the cochlea (44.6 ± 22.1%, *n* = 18 spiral ganglia) than at the base (12.8 ± 11.7%, *n* = 18 spiral ganglia, Student's *t‐*test with Welch's correction: *p* < 10^─3^ for all comparisons) (Figure 4B; Figure S9, Supporting Information). By contrast, only 2.1% of tdTomato‐positive cells contained *Prph* mRNA (*n* = 95 tdTomato‐positive cells in 13 spiral ganglia) and almost all the *Prph* mRNA containing neurons were tdTomato‐negative (94.8%, *n* = 39 neurons, Fisher's exact test *p* < 10^−5^) (Figure 4A,B). Therefore, Type II neurons do not express *Otogl*. For type I neurons, 46.1% of tdTomato‐positive neurons (*n* = 344 tdTomato‐positive neurons) were immunostained for Pou4f1, 26.6% (*n* = 498 tdTomato‐positive neurons) were immunostained for calbindin and 31.9% (*n* = 561 tdTomato‐positive neurons) for calretinin (24 cochlear sections, 3 animals). However, most Pou4f1‐positive (87.2%), calbindin‐positive (90.8%), and calretinin‐positive (89.3%) type I neurons were not tdTomato‐positive (Figure 4B). Thus, ≈46% of tdTomato‐positive neurons were type IC neurons thought to correspond to low SR neurons.

### 2.4 *Otogl* ^+/−^ Mutant Mice Display Auditory Nerve Dysfunctions

**Figure 5:**
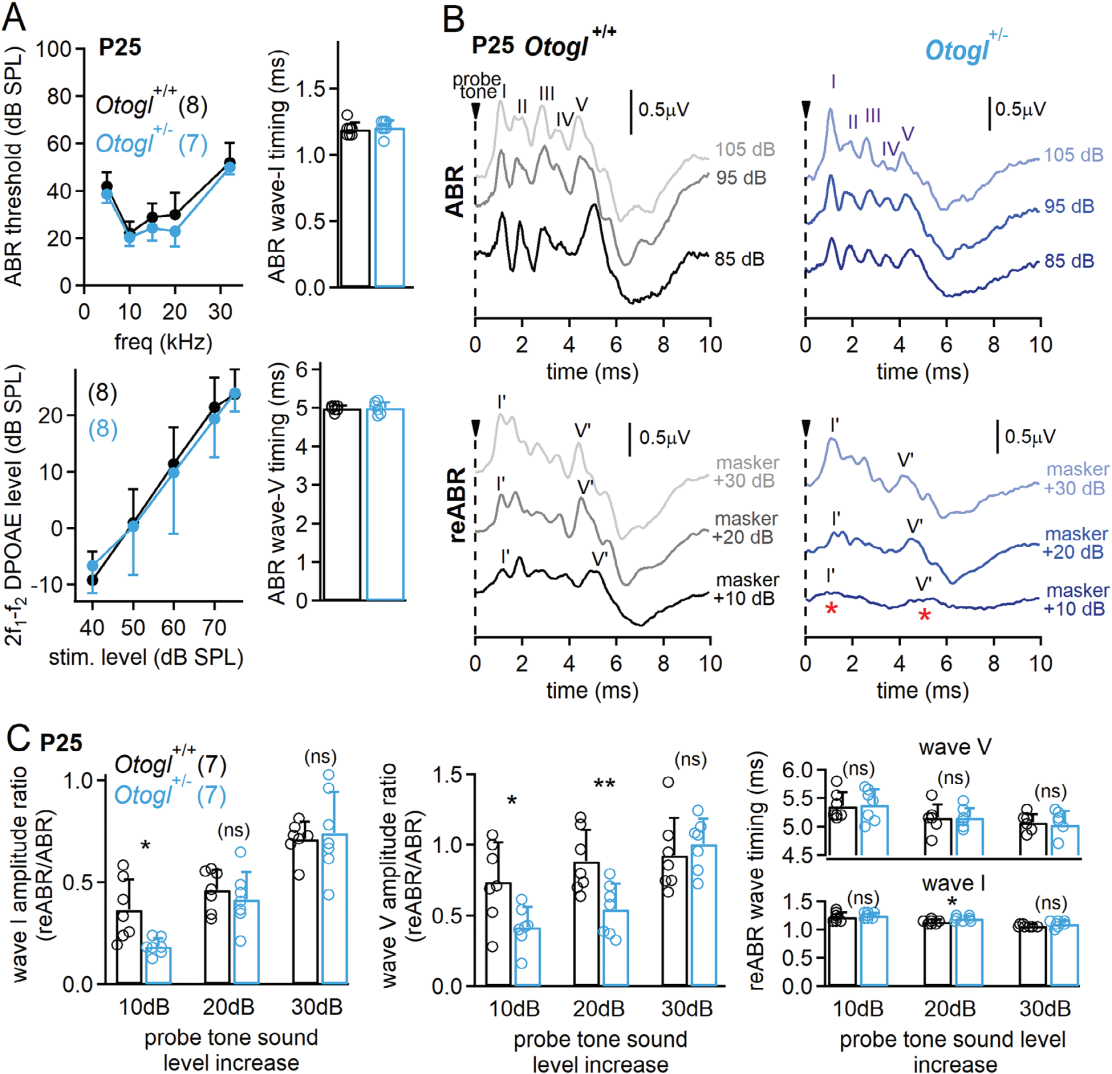
Elevated re‐ABR thresholds in *Otogl* ^+/−^ mutant mice. A) Graphs showing ABR thresholds (top left) and DPOAE levels (bottom left) in *Otogl* ^+/−^ and *Otogl* ^+/+^ mice on P25. Bar graphs showing the timing of ABR waves I (top right) and V (bottom right) in *Otogl* ^+/−^ and *Otogl* ^+/+^ mice. B) Example traces of ABR recordings in *Otogl* ^+/+^ (top left) and *Otogl* ^+/−^ (top right) mice on P25 in response to 10 kHz pure tones at 85, 95 and 105 dB. Example traces of reABR recordings obtained from *Otogl* ^+/+^ (bottom left) and *Otogl* ^+/−^ (bottom right) mice on P25 mice after increasing the 10 kHz probe tone by 10, 20, and 30 dB, after masking the classical ABR signal obtained in response to a 10 kHz probe tone at 75 dB with a ≈70 dB masker noise. Red asterisks indicate the almost total absence of re‐emerging wave I and V signals when the 10 kHz probe tone is increased by 10 dB. C) Bar graphs showing the wave I amplitude ratio (reABR/ABR) (left), the wave V amplitude ratio (middle), and the reABR wave timing (right) as a function of probe tone sound level increase. Numbers in brackets indicate the number of animals tested. ns, non‐significant.

*Otogl* ^+/−^ mutant mice emerged as a relevant model for studying the mechanisms underlying auditory hyperexcitability as they present the audiogenic seizure phenotype with no auditory threshold deficit.^[^ ^31^ ^]^ Measurements of distortion product acoustic emissions (DPOAEs), which probe OHC function, ABRs, and ABR wave I amplitude and timing were similar in *Otogl* ^+/−^ and *Otogl* ^+/+^ mice on P25 (Student's *t‐*test with Welch's correction, *p* > 0.1 for all statistical comparisons) (**Figure** **5**A), suggesting that high SR neurons, which drive most of the auditory response around the auditory threshold,^[^ ^43^ ^]^ were unaffected by monoallelic *Otogl* inactivation.

We assessed the function of low SR neurons in *Otogl* ^+/−^ mice with the recently developed non‐invasive reemerging ABRs (reABRs) technique.^[^ ^44^ ^]^ This paradigm is based on the saturation of high SR and supposedly most medium SR neurons with a masker, while measuring ABRs at sound levels at which low SR neurons remain far from saturation, such that the ABR signal recorded corresponds mostly to the activity of low SR neurons. In both *Otogl* ^+/−^ and *Otogl* ^+/+^ mice, the ABRs elicited at 75 dB sound pressure level (SPL) (i.e. ≈50 dB above the ABR threshold) by a tone pip at 10 kHz, the frequency used to elicit audiogenic seizures, were first masked by a broadband noise masker at ≈70 dB, flattening the ABR curve. An extra 10 dB increase in the probing tone pip resulted in reABR signals indicating an impairment principally in the activity of low SR neurons in *Otogl* ^+/−^ mice (Figure 5B). To compare the contribution of low SR neurons between mice, we normalized the measured reABR amplitude by the ABR amplitude. The reABR/ABR amplitude ratios of *Otogl* ^+/−^ mice were 47% lower for wave I and 43% lower for wave V than those of *Otogl* ^+/+^ mice (*p* = 0.02 for both comparisons, Student's *t‐*test with Welch's correction) (Figure 5C). The reABR timings of waves I and V were unaffected (*p* > 0.44 for both comparisons, Student's *t*‐test with Welch's correction). A further 10 dB increase in the probing tone (to reach a 20 dB increase) resulted in a wave I reABR/ABR amplitude ratio in *Otogl* ^+/−^ mice similar to that of *Otogl* ^+/+^ mice (*p* = 0.51, Student's *t*‐test with Welch's correction). However, the reABR/ABR wave V amplitude ratio remained 39% lower than that of *Otogl* ^+/+^ mice (*p* = 0.009). Finally, increasing the probing tone by 30 dB resulted in wave I and V reABR/ABR ratios that were similar between *Otogl* ^+/−^ and *Otogl* ^+/+^ mice (*p* > 0.53 for both comparisons) (Figure 5C). ReABRs were then recorded on P40–P45 when *Otogl* ^+/−^ mice were no longer prone to seizures. Both wave I and wave V reABR/ABR ratios were similar in *Otogl* ^+/−^ and *Otogl* ^+/+^ mice (*p* > 0.26 for both comparisons) (Figure S10, Supporting Information). This observation, jointly with the previous one at an earlier audiogenic seizure‐prone stage, strongly suggests a link between the audiogenic seizure phenotype and a defective reABR/ABR ratio. The wave I results suggest that an extra +10–20 dB are needed to lead to a normal activation of low SR neurons in *Otogl* ^+/−^ mice compared to *Otogl* ^+/+^ mice. In addition, the persistent wave V abnormality for tone pips that reemerge +20–30 dB above the masked situation suggests that auditory nerve deficits have more persistent effects in superior auditory relays. In conclusion, *Otogl* mutant mice display an auditory neuropathy caused by a weaker activation of high‐threshold low SR neurons.

### 2.5 Myelination of Auditory Nerve Neurons is Normal in *Otogl* ^+/−^ Mutant Mice

**Figure 6:**
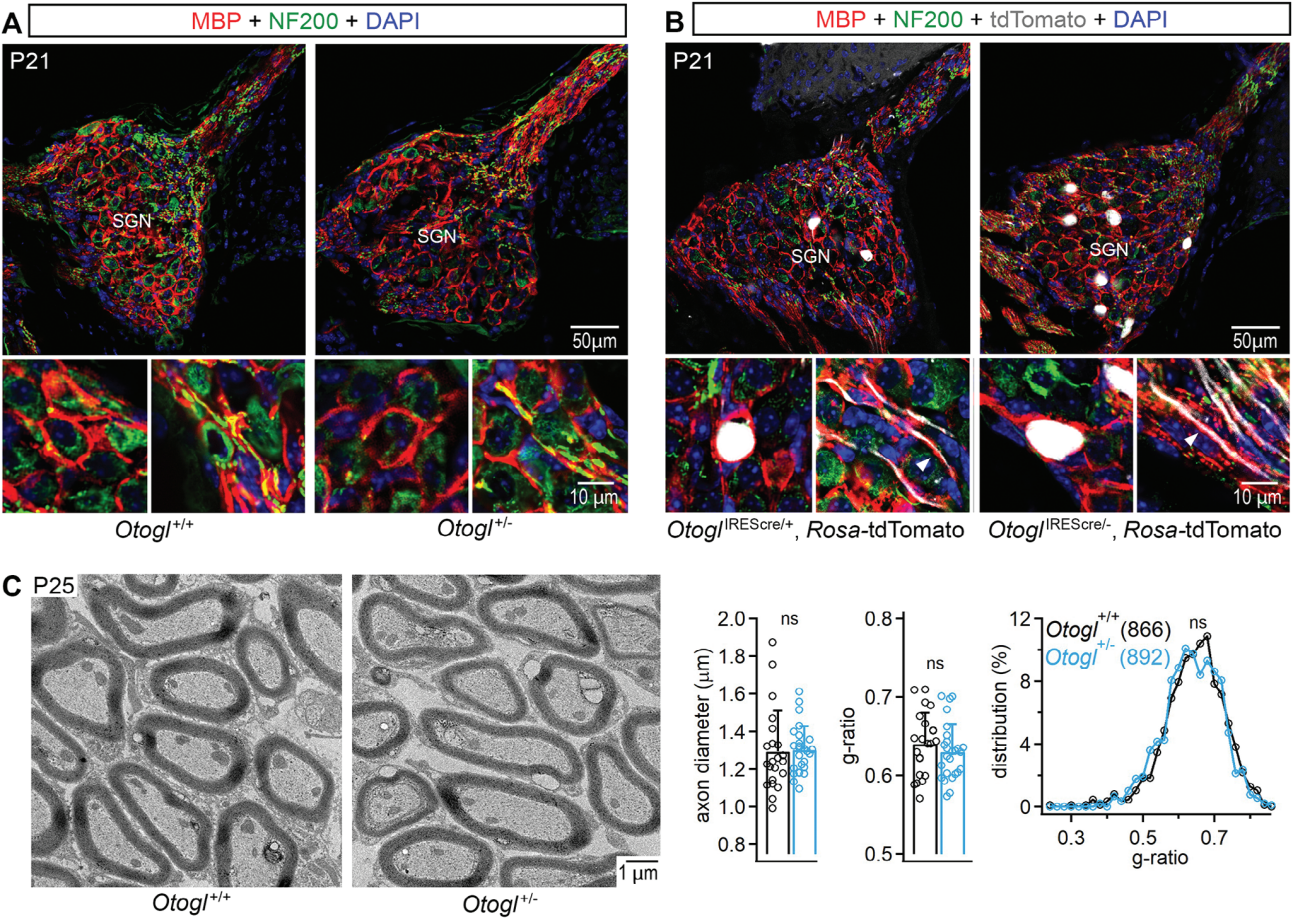
Myelination is not altered in *Otogl* ^+/−^ mutant mice. A) Cochlear spiral ganglia section of P21 *Otogl* ^+/+^ and *Otogl* ^+/−^ mice immunostained for MBP and the neurofilament protein NF200 (top) with enlarged views showing details of SGN cell bodies (bottom left) and axons (bottom right) for each genotype. B) Cochlear spiral ganglia section of P21 *Otogl* ^IREScre/+^:*Rosa‐*tdTomato and *Otogl* ^IREScre/−^:*Rosa‐*tdTomato mutant mice immunostained for MBP and NF200 (top) with enlarged views showing details of cell bodies (bottom left) and axons (bottom right) of tdTomato‐positive neurons (white) for each genotype. Arrowheads show examples of tdTomato‐positive axons (white) surrounded by MBP staining (red). C) Representative transmission electron microscopy acquisitions of P25 *Otogl* ^+/+^ and *Otogl* ^+/−^ axons of spiral ganglion neurons (left). Bar graphs showing the axon diameter and the g‐ratio of SGNs, and distribution of g‐ratios in analysed neurons (right). Numbers in brackets indicate the number of neurons analyzed. ns, non‐significant.

We then investigated whether myelin deficits, a potential cause of auditory neuropathies,^[^ ^45^, ^46^, ^47^ ^]^ could underlie the auditory nerve dysfunctions in *Otogl* ^+/−^ mutant mice. Cochleae from *Otogl* ^+/+^ and *Otogl* ^+/−^ mice were immunostained with antibodies directed against myelin basic protein (MBP), a key structural protein involved in the stability and integrity of the myelin sheath.^[^ ^45^, ^48^ ^]^ SGNs from *Otogl* ^+/+^ and *Otogl* ^+/−^ mice displayed apparently normal myelin sheaths both in the neurosensory epithelium and the spiral ganglia (**Figure** **6**A). In addition, tdTomato‐positive SGNs from *Otogl* ^IREScre/−^:*Rosa‐*tdTomato cochlear sections, in which the *Otogl* promoter was activated on a heterozygous *Otogl* background sufficient to induce audiogenic seizures, also had an apparently normal myelin sheath based on MBP staining (Figure 6B). Cochlear sections from *Otogl* ^+/+^ and *Otogl* ^+/−^ mice were then processed for transmission electron microscopy to carry out morphological analysis of myelin. Comparison of the axonal diameter and g‐ratio, the ratio between the inner axonal diameter and the total neuron outer diameter, revealed no statistically significant difference (*p* = 0.88 and *p* = 0.36 for axon diameter and g‐ratio, respectively; Student's *t*‐test with Welch's correction; Figure 6C) between *Otogl* ^+/+^ (22 images captured from 4 animals) and *Otogl* ^+/−^ mice (26 images captured from 4 animals). Notably, the distribution of g‐ratios between neurons from *Otogl* ^+/+^ and *Otogl* ^+/−^ mutant mice were not significantly different (*p* = 0.78; two‐sample Kolmogorov‐Smirnov test). Together, these observations suggest that otogelin‐like has neither a paracrine nor an autocrine role in SGN myelination.

### 2.6 Synaptic Ribbon Number and Exocytosis are not Altered in the IHCs of *Otogl* Mutant Mice

**Figure 7:**
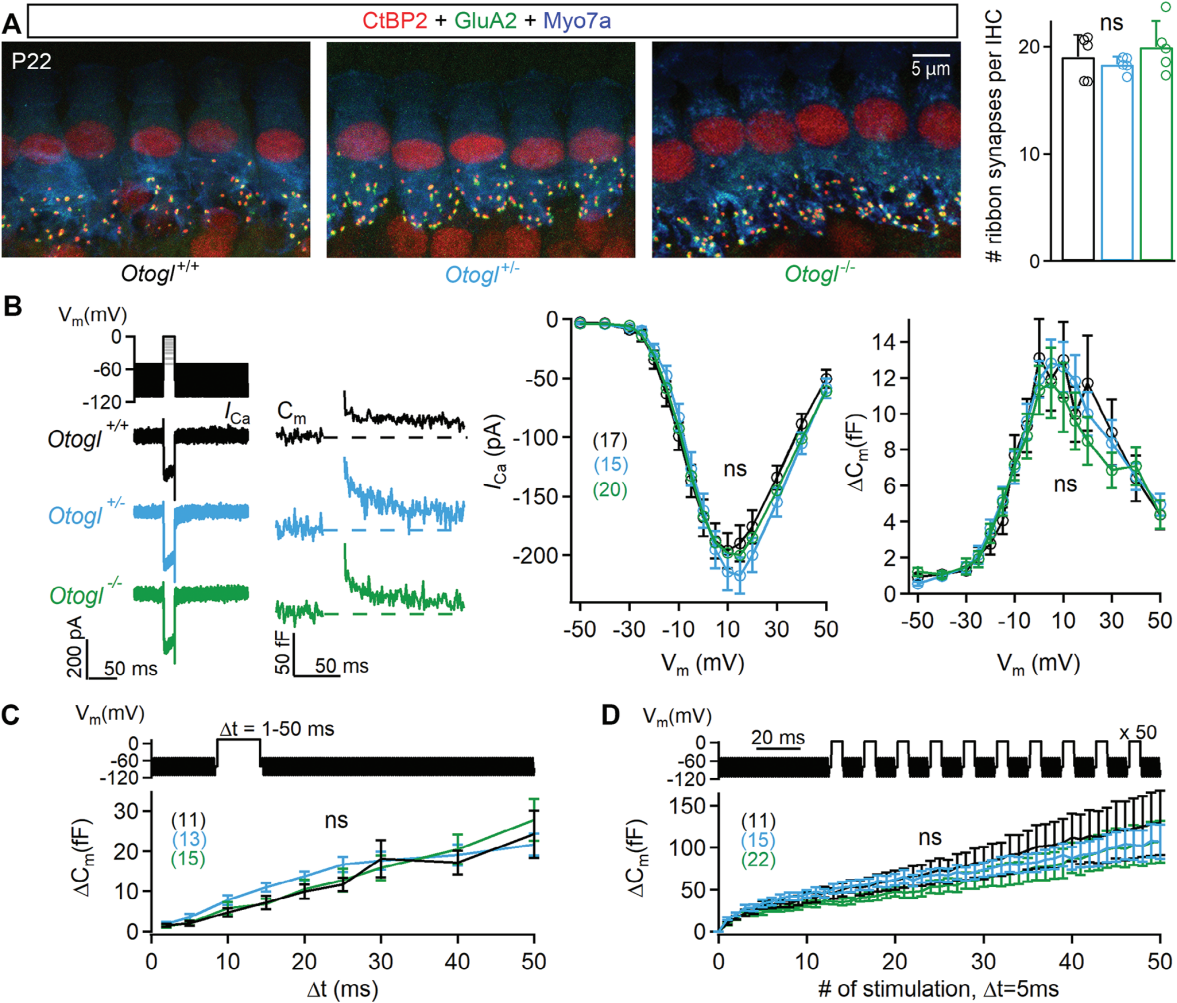
Ribbon synapse functioning is not altered in *Otogl* mutant mice. A) Z‐projections of IHCs from cochlear whole mounts from P22 *Otogl* ^+/+^, *Otogl* ^+/−^, and *Otogl* ^−/−^ mice (left). Bar graphs showing the number of ribbon synapses per IHC in *Otogl* ^+/+^, *Otogl* ^+/−^, and *Otogl* ^−/‐^ mice (right). B) Protocol used to depolarise IHCs from −95 mV to potentials between −65 mV to +35 mV, with examples of Ca^2+^ currents (*I*_Ca_) (left) and the corresponding C_m_ traces (right) for P25–P35 *Otogl* ^+/+^, *Otogl* ^+/−^, and *Otogl* ^−/−^ IHCs after 20 ms of depolarisation to −10 mV (left). Mean Ca^2+^ current amplitudes (*I*_Ca_) (middle) and ΔC_m_ (right) for P25–P35 *Otogl* ^+/+^, *Otogl* ^+/−^, and *Otogl* ^−/−^ IHCs after 20 ms of depolarisation to potentials between −65 mV and +35 mV. C) Protocol used to depolarise IHCs from −95 mV to −10 mV for voltage steps of various durations from 2 to 50 ms (top). Kinetics of Ca^2+^‐dependent exocytosis in P25–P35 *Otogl* ^+/+^, *Otogl* ^+/−^, and *Otogl* ^−/−^ IHCs for voltage steps of 2 ms to 50 ms. Mean ΔC_m_ is plotted against the duration of depolarisation to −10 mV (Δt). For the 2 and 5 ms depolarizations, the three repeated recordings were averaged, to increase the signal‐to‐noise ratio. D) Protocol used to elicit a train of 50 successive short depolarisations (duration 5 ms, interpulse interval 10 ms) to −10 mV (top) and plots of mean cumulative ΔC_m_ as a function of stimulus number in response to the train of 50 successive short depolarisations in P25‐P35 *Otogl* ^+/+^, *Otogl* ^+/−^, and *Otogl* ^−/−^ IHCs (bottom). For each depolarisation, ΔC_m_ was evaluated by averaging only the last 3 ms of C_m_ values for each interstimulus interval, to prevent contamination with the initial peaks. Numbers in brackets indicate the number of patched IHCs. ns, non‐significant.

The auditory neuropathy in *Otogl* ^+/−^ mice may result from an intrinsic deficit in low SR neurons,  a deficit of synapses between IHCs, which also express *Otogl*, and SGNs, or both. We investigated the presynaptic role of *Otogl* by counting the total number of ribbons — the homophilic structure around which IHC presynaptic vesicles are tethered^[^ ^49^, ^50^, ^51^ ^]^ — in *Otogl* mutant mice, using antibody labeling directed against the presynaptic ribbon marker CtBP2 and the postsynaptic glutamate receptor subunit GluA2. Ribbon synapse numbers were similar between the IHCs of *Otogl* ^+/+^, *Otogl* ^+/−^ and *Otogl* ^−/−^ mice (**Figure** **7**A) (*p* > 0.21 for all comparisons, Student's *t*‐test with Welch's correction).

We then probed the exocytosis of IHCs in *Otogl* mutant mice by monitoring depolarisation‐evoked membrane capacitance changes (ΔC_m_) as a proxy for vesicle fusion.^[^ ^50^ ^]^ Ca^2+^ currents (*I*_Ca_) and the corresponding ΔC_m_ were recorded in response to depolarisations of various amplitudes (from a holding membrane potential of −95 mV to potentials between −65 mV and +35 mV), each lasting 20 ms, during which synaptic exocytosis mostly reflected the fusion of vesicles from the readily releasable pool presumed to lie close to the presynaptic membrane. *I*_Ca_ and ΔC_m_ amplitudes were similar between *Otogl* ^+/+^, *Otogl* ^+/−^, and *Otogl* ^−/−^ mice (*p* = 0.99, two‐way ANOVA, interaction *I*_Ca_ x group, F(30,796) = 0.38; *p* = 0.95, two‐way ANOVA, interaction ΔC_m_ x group, F(30,706) = 0.35) (Figure 7B). Moreover, ΔC_m_ did not differ between *Otogl* ^+/+^, *Otogl* ^+/−^, and *Otogl* ^−/−^ mice when IHCs were subjected to brief (2 to 50 ms) depolarizations to −10 mV, suggesting that *Otogl* mutations do not affect the release kinetics of the readily releasable pool (*p* = 0.95, two‐way ANOVA, interaction Δt x group, F(16,329) = 0.50) (Figure 7C). We also probed the recycling pools of vesicles^[^ ^52^ ^]^ thought to replenish the readily releasable pool upon sustained release, by periodic stimulation with 50 short (5 ms long) depolarizations to −10 mV 10 ms apart.^[^ ^53^, ^54^ ^]^ The responses obtained were normal in *Otogl* ^+/−^ and *Otogl* ^−/−^ mice (*p* = 1.0, two‐way ANOVA, interaction ΔC_m_ x group, F(102,2338) = 0.14) (Figure 7D). In conclusion, no structural or functional presynaptic deficit was detected in *Otogl* ^+/−^ or *Otogl* ^−/−^ mice, suggesting that an intrinsic deficit of SGNs was responsible for the abnormal reABRs.

### 2.7 Conditional Inactivation of *Otogl* in Spiral Ganglion Neurons Reproduces Auditory Nerve Dysfunction

**Figure 8:**
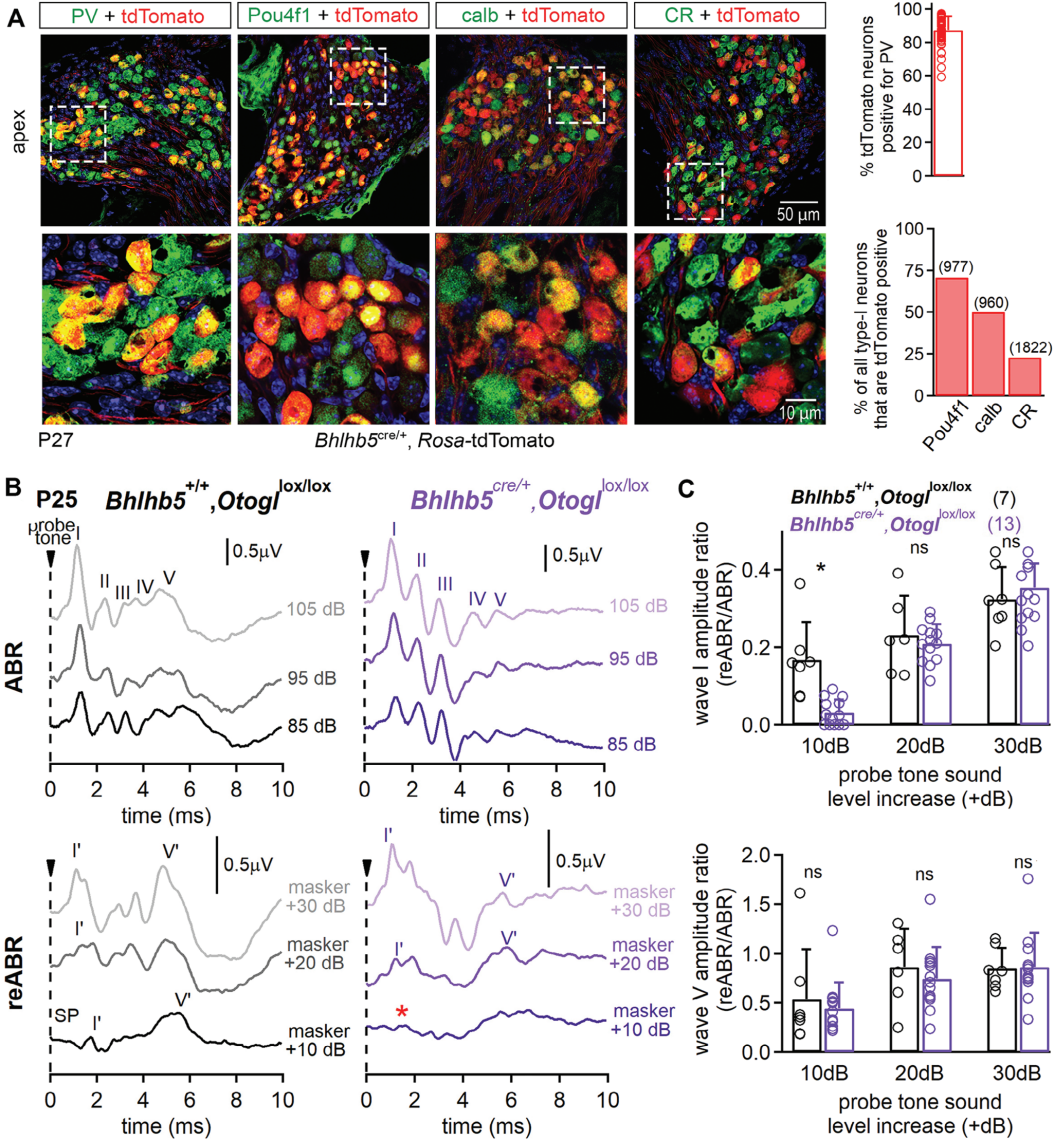
Elevated re‐ABR thresholds in *Otogl* conditional knock‐out mice. A) Z‐projections of apical cochlear spiral ganglia cross‐sections from a P27 *Bhlhb5* ^cre/+^:*Rosa‐*tdTomato mouse immunostained for PV, Pou4f1, calb, or CR (top) with enlarged views of the insets showing details of SGNs (bottom). Bar graphs showing the percentage of tdTomato‐positive neurons also positive for PV (top) and the percentage of tdTomato‐positive neurons also positive for Pou4f1, calb, or CR (bottom). Cell nuclei are stained in blue (DAPI). B) Example traces of ABR recordings from P25 control *Bhlhb5* ^+/+^:*Otogl* ^lox/lox^ (top left) and *Bhlhb5* ^cre/+^:*Otogl* ^lox/lox^ (top right) in response to 10 kHz pure tones at 85, 95, and 105 dB. Example traces of reABR recordings obtained from P25 *Bhlhb5* ^+/+^:*Otogl* ^lox/lox^ (bottom left) and *Bhlhb5* ^cre/+^:*Otogl* ^lox/lox^ (bottom right) mice, after increasing the 10 kHz probe tone by 10, 20, and 30, with masking of the classical ABR signal obtained in response to a 10 kHz probe tone at 75 dB with a ≈70 dB masker noise. The red asterisk indicates the almost total absence of a re‐emerging wave I signal when the 10 kHz probe tone is increased by 10 dB. C) Bar graphs showing the wave I amplitude ratio (reABR/ABR) (top) and the wave V amplitude ratio (bottom) as a function of probe tone sound level increase in P25 *Bhlhb5* ^+/+^:*Otogl* ^lox/lox^ and *Bhlhb5* ^cre/+^:*Otogl* ^lox/lox^ mice. Numbers in brackets indicate either the number of probed neurons (A) or tested animals (C). ns, non‐significant.

We investigated the intrinsic role of *Otogl* in the SGNs by crossing *Otogl* ^lox/lox^ mice with *Bhlhb5* ^cre/+^ knock‐in mice, to achieve conditional inactivation through cre expression under the control of the *Bhlhb5* promoter,^[^ ^55^ ^]^ which was active in SGNs but not in hair cells.^[^ ^56^, ^57^, ^58^ ^]^ Before the functional analysis of *Bhlhb5* ^cre/+^:*Otogl* ^lox/lox^ mice, we assessed the penetrance of cre expression in the different subtypes of type I neurons by generating *Bhlhb5* ^cre/+^:*Rosa‐*tdTomato mice. In these mice, tdTomato was expressed by only 48.5% of PV‐positive type I neurons (*n* = 3534 PV neurons, 50 sections, 3 animals), the distribution of these cells varying along the tonotopic axis (43% at the apex, 32.6% in the middle, and 24.4% at the base). However, 70.9% (*n* = 977) of Pou4f1‐positive neurons, thought to correspond to low SR neurons, were also positive for tdTomato, whereas only 50% of calb‐positive (*n* = 960 neurons) and 22.9% of CR‐positive (*n* = 1822) neurons also expressed tdTomato. The *Bhlhb5*‐cre driver line was therefore considered relevant for the conditional inactivation of genes in most type IC neurons (**Figure** **8**A; Figure S11, Supporting Information).

As expected, *Bhlhb5* ^cre/+^:*Otogl* ^lox/lox^ mice had similar ABR and DPOAE thresholds to *Bhlhb5* ^+/+^:*Otogl* ^lox/lox^ mice on P25, further confirming that the cre driver line was not active in the sensory epithelium, the organ of Corti (*p* > 0.25 for all comparisons, Student's *t*‐test with Welch's correction). However, no signs of audiogenic seizures were observed in *Bhlhb5* ^cre/+^:*Otogl* ^lox/lox^ (*n* = 42) and *Bhlhb5* ^+/+^:*Otogl* ^lox/lox^ mice (*n* = 34) between P22 and P30 (*p* = 0.9, Fisher's exact test). This result suggests that the conditional inactivation of *Otogl* in only a fraction of SGNs was insufficient to cause a susceptibility to audiogenic seizures. The reABR/ABR amplitude ratio for wave I was 82% smaller in *Bhlhb5* ^cre/+^:*Otogl* ^lox/lox^ mice than in control *Bhlhb5* ^+/+^:*Otogl* ^lox/lox^ mice (*p* = 0.01, Student's *t*‐test with Welch's correction) (Figure 8B,C), further confirming that the reABR/ABR wave I amplitude ratio deficit was intrinsic to SGNs and not linked to an IHC synaptopathy. However, reABR/ABR wave V ratios were similar in control and *Bhlhb5* ^cre/+^:*Otogl* ^lox/lox^ mice (*p* = 0.66, Student's *t*‐test with Welch's correction), suggesting that a decrease of this ratio was a marker of susceptibility to audiogenic seizures.

### 2.8 Central Auditory Consequences of Auditory Nerve Dysfunction in *Otogl* ^+/−^ Mice

**Figure 9:**
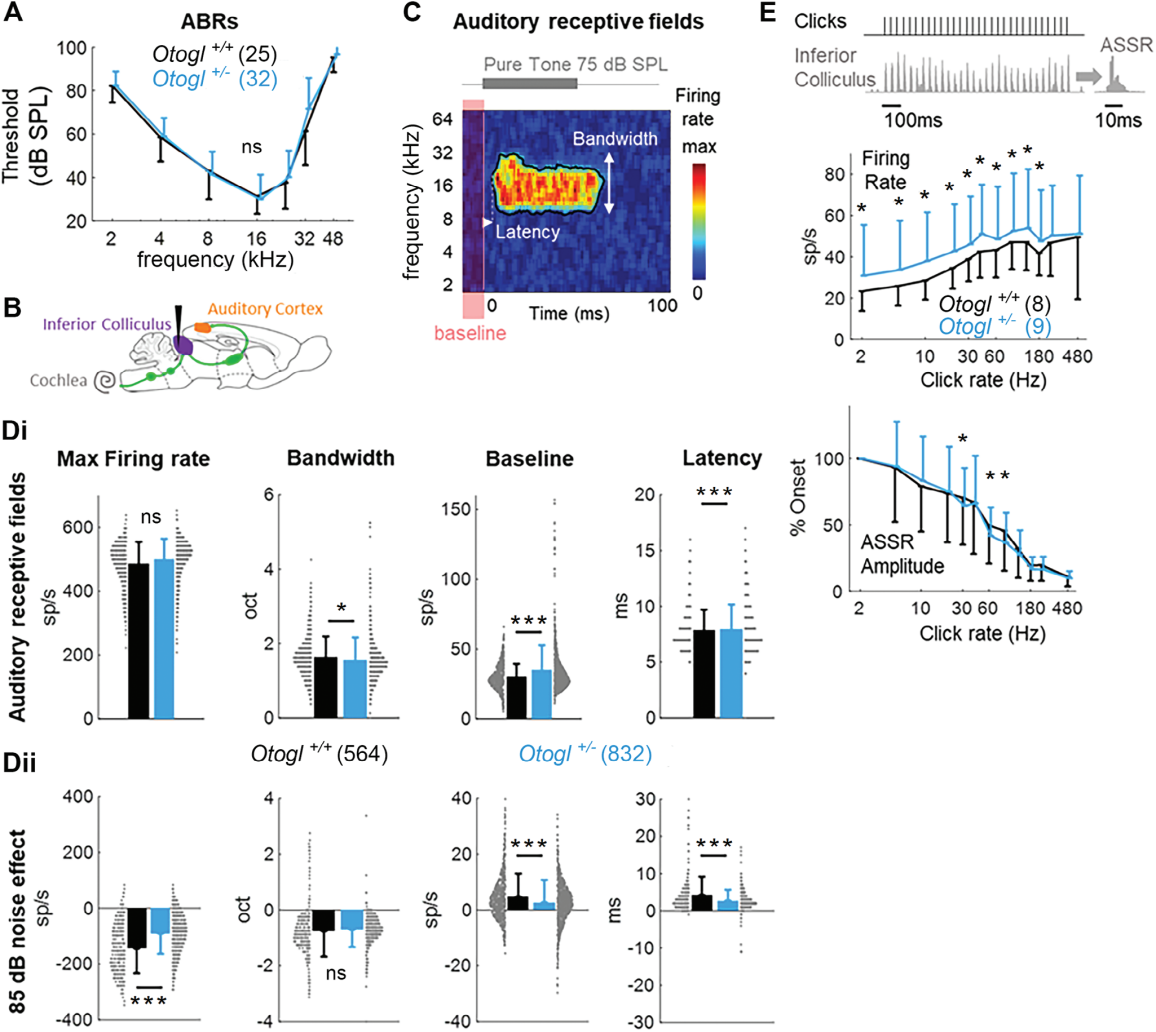
Central auditory processing in *Otogl* ^+/−^ mutant mice. A) Auditory thresholds determined from ABRs in the range 2–48 kHz in *Otogl* ^+/−^ and *Otogl* ^+/+^ mice. B) In vivo configuration for electrophysiological recordings with the implantation of a laminar microelectrode (black) in the inferior colliculus of 8 *Otogl* ^+/+^ and 9 *Otogl* ^+/−^ mice. C) Spectrotemporal auditory receptive fields (STRFs) of inferior colliculus neurons in response to a 50 ms pure tone measured as firing rate as a function of tone frequency and time. The neural response was characterized by extracting from the STRF the bandwidth of the significant response area, the latency of the neural response, the maximum evoked firing rate and the baseline activity level (average firing rate in the 10 ms preceding the tone stimulation) as illustrated on this individual example. Di) Bar graphs showing the parameter values for STRFs obtained from the response to 75 dB SPL pure tones in *Otogl* ^+/−^ and *Otogl* ^+/+^ mice. Distribution is indicated in grey. Dii) Bar graphs showing the variation of these parameters following the addition of background white noise at 85 dB SPL. Distribution is indicated in grey. E) Temporal processing in the inferior colliculus of *Otogl* ^+/−^ and *Otogl* ^+/+^ mice. Example of a neural response to a 1 s click train at 30 Hz (top). ASSR was defined as the averaged response to each click (top). Graphs showing the mean firing rate over 1 s as a function of click rate (middle) and ASSR maximum firing rate relative to that of the first click (onset response), as a function of click rate (bottom). The latter function illustrates the progressive decrease in phase‐locking ability with increasing click rate. Numbers in brackets indicate the number of either animals tested (A, E) or recorded neurons (D). ns, non‐significant.

The susceptibility of *Otogl* ^+/−^ mice to audiogenic seizures and the impaired activation of low SR neurons in these mice suggest a possible connection between low SR neurons and inhibitory neuronal circuits, or defects of mechanisms protecting the auditory system against loud sound exposures in *Otogl* ^+/−^ mice. We investigated the possible connection between low SR neurons and inhibitory neuronal circuits, by studying central auditory processing in the inferior colliculus, which is thought to be involved in audiogenic seizure susceptibility,^[^ ^59^ ^]^ in anaesthetized *Otogl^+/+^* and *Otogl* ^+/−^ mice with implanted laminar electrodes (**Figure** **9**A,B). ABR thresholds were normal in *Otogl* ^+/−^ and *Otogl* ^+/+^ mice (*p* = 0.09, two‐way ANOVA, interaction between group and frequency, F(6,378) = 1.82). Auditory spectrotemporal receptive fields (STRFs) (Figure 9C,Di) were only moderately affected in *Otogl* ^+/−^ mutant mice relative to *Otogl^+/+^* controls, with a slightly significant change in response latency (*p* = 7.10^−8^, nested two‐way ANOVA, group effect, animal factor nested in group, F(1,1379) = 29.5) and a 5.8% decrease in frequency bandwidth (*p* = 0.048, nested ANOVA, F(1,1379) = 3.9), with no change in maximum evoked firing rate (*p* = 0.06, nested ANOVA, F(1,1379) = 3.5). However, baseline activity was markedly higher by 17.5% (*p* = 10^−9^, nested ANOVA, F(1,1379) = 36,8), suggesting either an increase in excitation or a decrease in inhibition in the inferior colliculus of *Otogl* ^+/−^ mutant mice. With the addition of background white noise at 85 dB SPL, which typically leads to narrower, smaller, delayed STRFs accompanied by an increase in baseline activity (Figure 9Dii, black bars),^[^ ^60^ ^]^ the neurons of *Otogl* ^+/−^ mice were significantly less affected than those of *Otogl^+/+^* mice in terms of maximum evoked firing rate (*p* < 10^−10^, nested ANOVA, F(1,767) = 77.8), baseline activity (*p* = 3.10^−4^, nested ANOVA, F(1,1379) = 13) and latency (*p* = 3.10^−5^, nested ANOVA, F(1,767) = 17.7). This suggests that the suppressive effects of the neural response to noise were mitigated, consistent with a decrease in inhibition upstream from the inferior colliculus. We subsequently investigated the temporal processing of neurons with click trains as acoustic stimuli (Figure 9E). Consistent with the STRF‐related results, mean firing rate was 11 to 33% higher in *Otogl* ^+/−^ mice than in *Otogl* ^+/+^ mice for click rates between 2 and 180 Hz (*p* < 10^−10^, three‐way ANOVA, interaction between group and frequency, animal nested into group, F(11,13667) = 6.24, Figure 9E). In addition, fast click rates (30, 60 and 90 Hz) elicited a smaller auditory steady‐state response (ASSR) in *Otogl* ^+/−^ mice than in *Otogl* ^+/+^ mice, suggesting an impairment of phase‐locking in neurons (*p* < 10^−10^, three‐way ANOVA, interaction between group and frequency, animal nested into group F(11,13667) = 6.96, Figure 9E). Overall, the inferior colliculus neurons of *Otogl* ^+/−^ mice had a higher baseline activity and were more sensitive to acoustic stimuli but were less affected by background noise than the neurons of *Otogl* ^+/+^ mice. Based on the weaker activation of low SR neurons in *Otogl* ^+/−^ mutant mice, we suggest that the greater excitability of inferior colliculus neurons results from a decrease in inhibition rather than an increase in excitation.

### 2.9 The Middle the ear Muscle Reflex of *Otogl* ^+/−^ Mice is Defective

**Figure 10:**
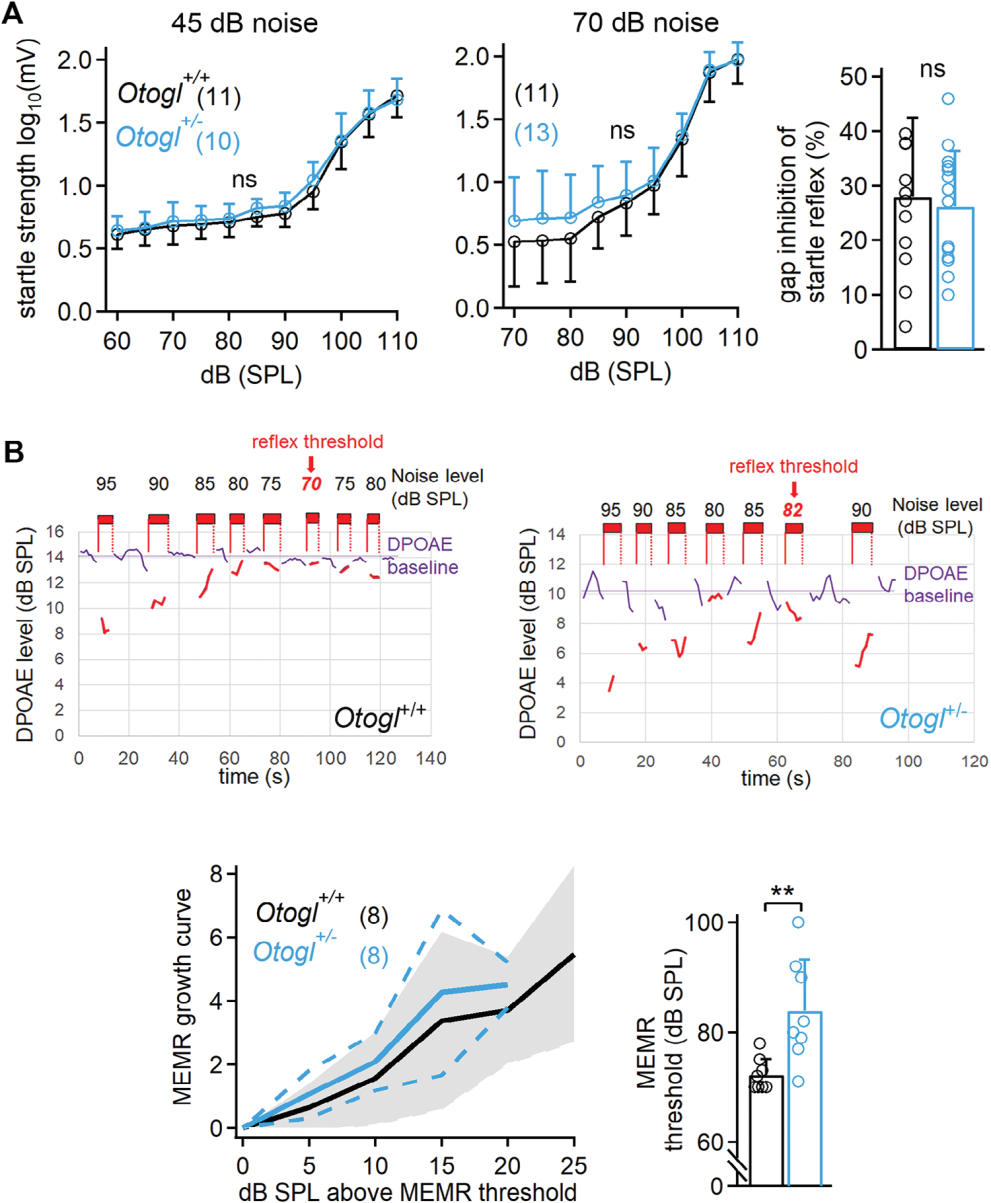
Abnormal MEMR in *Otogl* ^+/−^ mutant mice. A) Startle reflex amplitude as a function of the sound stimulus with a background of 45 dB white noise (left) and a background of 70 dB white noise (middle), and measurements of the gap inhibition startle reflex (right). B) Examples of DPOAE recordings in one the ear of *Otogl* ^+/+^ (top left) and *Otogl* ^+/−^ (top right) mice, in the intermittent presence of a contralateral filtered white noise stimulus. The noise was played at different amplitudes to elicit the MEMR, for determination of its activation threshold. Graph of the mean MEMR (±SD) curve as a function of the noise elicitor level above an individually measured reflex threshold (bottom left) and bar graph showing the mean activation threshold of the MEMR (bottom right). Numbers in brackets indicate the number of animals tested. ns, non‐significant.

We then investigated possible impairments of the auditory reflex circuits that protect the auditory system against some of the effects of loud sounds in *Otogl* ^+/−^ mutant mice. We first tested the startle reflex. The startle reflex did not differ between P18–P25 *Otogl* ^+/−^ and *Otogl* ^+/+^ mice probed in a quiet environment (*p* = 1, two‐way ANOVA, interaction SPL x group, F(10 253) = 0.14) or in the presence of a background noise masker of 70 dB SPL (*p* = 1, two‐way ANOVA, interaction startle x group, F(8 234) = 0.14), and no gap inhibition difference was detected during tests at 110 dB SPL in the presence of background noise (*p* = 0.72, Student's *t*‐test with Welch's correction) (**Figure** **10**A). We then probed the MEMR, a bilateral middle the ear acoustic reflex causing contraction of the stapedial and tensor tympani muscles attached to the stapes and malleus, respectively, thereby decreasing the level of sound reaching the inner ear.^[^ ^61^ ^]^ Because of the decrease in middle the ear admittance, this reflex also leads to a change in the phase of the DPOAE signal and a decrease in its amplitude.^[^ ^62^ ^]^ These concomitant changes can be used to determine the MEMR threshold, which was 72 ± 3 dB SPL in wild‐type *Otogl* ^+/+^ mice (Figure 10B). In *Otogl* ^+/−^ mice, the MEMR threshold was abnormally high, at 84 ± 9 dB SPL (*p* = 0.005, Mann‐Whitney test). Despite the threshold shift, the mean growth in MEMR induced DPOAE changes with white noise elicitor level above threshold was identical in *Otogl* ^+/−^ and *Otogl* ^+/+^ mice (Figure 10B). Together, the concomitant increases in activation threshold for a population of low SR neurons and MEMR strongly suggest that low SR neurons drive this reflex circuit.

## 3 Discussion

By investigating the mechanisms underlying audiogenic seizures in a mouse model for a genetic form of deafness, we uncovered that *Otogl* monoallelic inactivation results in an auditory neuropathy. Several of our findings show that *Otogl* is a marker of a population of type I SGNs at their very early stage of embryonic formation. TdTomato expression was detected in developing SGNs in *Otogl* ^IREScre/+^:*Rosa‐*tdTomato mice as early as E12.5. *Otogl* mRNA was transiently detected in these neuroblasts soon after their delamination in the otic vesicle, but before the start of their migration. In addition, the number of tdTomato‐positive SGNs in *Otogl* ^IREScre/+^:*Rosa‐*tdTomato mice remained stable after P0. These SGNs spanned the three subtypes of type I neurons: type IC (≈46%) and IB (≈27%), which may arise from the same prenatal developmental lineage in mice,^[^ ^12^, ^63^ ^]^ and type IA (≈32%). Although the three molecular subtypes were present, the morphology of synaptic contacts made by tdTomato‐positive neurons in the CN of *Otogl* ^IREScre/+^:*Rosa‐*tdTomato mice was homogeneous. Notably, none of these neurons formed an endbulb of Held giant synapse with principle cells in the CN; instead they only formed small bouton synapses. This suggests that a given SGN subtype as presently defined functionally^[^ ^5^, ^6^ ^]^ or molecularly^[^ ^8^, ^9^, ^10^ ^]^ may actually be subdivided further based on the synaptic contacts it forms in the CN as endbulbs of Held are thought to arise from all three types of type I SGNs.^[^ ^64^ ^]^ Consistent with the findings for intracellular injections in cats showing that low SR neurons send projections to the neuropil between the ventral and dorsal CN,^[^ ^65^ ^]^ most of the tdTomato‐positive neurons in *Otogl* ^IREScre/+^:*Rosa‐*tdTomato mice were in the ventral rather than the dorsal CN. Early *Otogl* expression thus defines an early committed subpopulation of SGNs principally of type IC forming exclusively bouton‐like synapses in the CN.

*Otogl* ^+/−^ mutant mice emerged as the ideal model for investigating the retrocochlear consequences of *Otogl* mutations because they are susceptible to audiogenic seizures but have no peripheral deficit with normal OHC amplification (Figure 5A and reference^[^ ^31^ ^]^). The reABRs of these mice revealed the need for ≈10–20 dB louder sounds to activate low SR neurons as much as in controls. This deficit is unlikely to result from a presynaptic defect because IHC morphology and exocytosis rates were normal in *Otogl* ^+/−^ and *Otogl* ^−/−^ mutant mice, and the conditional biallelic inactivation of *Otogl* in SGNs reproduced a similar activation deficit in low SR neurons. In contrast, ABR thresholds and wave I amplitude, which result from the synchronous activity of high and medium SR neurons,^[^ ^43^ ^]^ were indistinguishable between *Otogl* ^+/+^ and *Otogl* ^+/−^ mice, further demonstrating the greater importance of otogelin‐like in low SR neurons.

What role does otogelin‐like play in SGNs? *Otogl* encodes a large protein of the tectorial membrane related to secreted epithelial mucins.^[^ ^31^, ^32^, ^66^ ^]^ Based on the normal timings of ABR waves I and V, and normal myelin thickness in *Otogl* ^+/−^ mutant mice, a role for otogelin‐like in myelination is unlikely. Furthermore, although we cannot formally rule out the existence of an unidentified transmembrane domain, the *Otogl* transcripts reported so far suggest that otogelin‐like is an extracellular protein,^[^ ^67^ ^]^ which could play autocrine and/or paracrine roles in SGN development. Otogelin‐like produced by cell types of the organ of Corti is very unlikely to play a role in SGN development because the abnormal reABR phenotype was also observed with the *Bhlhb5*‐cre driver line, which does not express the cre in the organ of Corti. We therefore propose that the auditory nerve deficit is due to otogelin‐like produced by SGNs themselves.

SGNs expressing *Otogl* may be part of a specific auditory circuit involving a fraction of low SR neurons that could be involved in auditory hyperexcitability. Audiogenic seizures have been previously linked to a dysfunction of the auditory pathways inducing an abnormal response of the inferior colliculus leading to the spread of the seizure to other brain networks.^[^ ^59^ ^]^ Audiogenic seizures have been associated with auditory nerve deficits in *Gipc3* mutant mice, in which the magnitude and temporal attributes of the ABR wave I amplitude are correlated with susceptibility to audiogenic seizures.^[^ ^68^ ^]^ Inhibitory neuron deficits have also been described in the auditory cortex of mouse models of Usher syndrome susceptible to audiogenic seizures, including *Cdhr15* ^+/−^, *Cdhr23* ^+/−^, and *Adgrv1* ^−/−^ mice.^[^ ^30^ ^]^ Our results support the existence of another mechanism by which the audiogenic seizure phenotype in *Otogl* ^+/−^ mutant mice results from defective low SR neurons involved in loud sound detection, confirming our inference that low SR neurons activate neural circuits controlling auditory excitability. In comparison with control wild‐type mice, *Otogl* ^+/−^ mutant mice had a concomitant difference of ≈10–20 dB in sound levels required for allowing a given activation of their low SR neurons and an elevation of their MEMR reflex threshold, supporting previous suggestions that low SR neurons may control this reflex.^[^ ^24^, ^69^, ^70^, ^71^ ^]^ Loud sounds (≈80–100 dB) would result in full stimulation of the auditory system of *Otogl* ^+/−^ mice, through the saturated activation of high and medium SR neurons in the absence of sound‐attenuating reflexes. This hyperexcitability may be sufficiently strong to render *Otogl* ^−/−^ mice even more prone to audiogenic seizures than *Otogl* ^+/−^ mice, despite their moderate hearing loss. Furthermore, the neurons expressing *Otogl* may activate other inhibitory circuits of the auditory system, as reflected by the higher levels of baseline activity for inferior colliculus neurons in *Otogl* ^+/−^ mice and the lack of reactivity of these neurons in the presence of background noise relative to inferior colliculus neurons in *Otogl* ^+/+^ mice. These findings suggest that low SR neurons expressing *Otogl* play a major role in controlling the activity of the auditory system in cases of exposure to loud sounds by activating the MEMR and inhibitory circuits of the central auditory system. Indeed, decreases in MEMR activity are widely recognised as a useful biomarker of auditory neuropathy^[^ ^72^ ^]^ that might be specifically linked to low SR neuron dysfunction.^[^ ^73^ ^]^ As for auditory hyperexcitability, tinnitus or hyperacusis in patients with normal pure‐tone audiograms have been tentatively attributed to hidden cochlear synaptopathy affecting low SR neurons,^[^ ^26^, ^74^ ^]^ similar to that histologically proven in animal models displaying a full recovery of auditory thresholds after a single overexposure to a loud sound.^[^ ^19^ ^]^

In human subjects at risk of overexposure to loud sounds, but with normal pure‐tone audiograms and no clinically identifiable disorder, the concept of hidden low SR neuron dysfunction is difficult to substantiate. The difficulty of these individuals with understanding speech in noisy conditions is not inconsistent with a neuropathy diagnosis, but has an ambiguous clinical significance^[^ ^75^ ^]^ in the absence of hallmarks of auditory neuropathies, such as flat ABRs, which require all SGN subgroups to be affected.^[^ ^3^ ^]^ The present results indicate that genetics may be particularly suitable to uphold the concept of hidden low SR neuron dysfunction. More than 140 isolated (non‐syndromic) and ≈ 400 syndromic genetic forms of deafness,^[^ ^34^, ^76^ ^]^ as well as about a dozen genetic auditory neuropathies and synaptopathies have been described.^[^ ^77^ ^]^ However, these genetic neuropathies are generally associated with congenital/progressive moderate to severe hearing loss or a drastic loss of SGNs, precluding studies of the auditory circuits associated with each subtype of SGNs. Heterozygous *Otogl* ^+/−^ mutant mice have a mild SGN functional phenotype, with preserved auditory thresholds. Studies of *OTOGL* ^+/−^ humans may therefore be invaluable to unveil new possibilities for identifying a specific auditory circuit involved in sound hypersensitivity, and to unravel the links between auditory neuropathy and auditory hyperexcitability.

## 4 Experimental Section

### Animals

Animal experiments were performed in accordance with French and European regulations for the care and protection of laboratory animals (EC Directive 2010/63, French Law 2013–118, February 6, 2013), under authorisation from Institut Pasteur's Ethics Committee for Animal Experimentation.

*Bhlhb5* ^cre/+^ knock‐in recombinant mice were provided by Lin Gan, University of Rochester.^[^ ^55^ ^]^ The *Rosa*‐tdTomato Ai9 mouse line was obtained from Jackson Laboratories. The *Otogl* (MGI:3647600) conditional and knock‐out mouse lines (*Otogl* ^tm1Ugds/tm1Ugds^ mice, referred to here as *Otogl* ^−/−^ mice) had been described elsewhere.^[^ ^31^ ^]^ The *Otogl* ^IREScre/+^ mice were established at the MCI/ICS (Mouse Clinical Institute – Institut Clinique de la Souris ‐, Illkirch, France; http://www‐mci.u‐strasbg.fr). The targeting vector was constructed as follows: The IRES‐CRE‐F3‐ERT2‐F3 and Rox‐NeoR‐pPGK‐Rox sequences were inserted behind the STOP codon of *Otogl* in a construct including exon 58 of *Otogl* (ENSEMBL ENSMUSG00000091455). The targeted construct was introduced into embryonic stem cells from C57BL/6N mice by electroporation. Stem cells carrying the desired construct were injected into blastocysts from BALB/cN mice. Chimeras were bred with a C57BL/6N Dre deleter female (developed at PHENOMIN‐ICS, unpublished) that shows maternal contribution, to delete the Rox‐pPGK‐NeoR‐Rox selection cassette, generating the *Otogl* ^IREScreERT2/+^ mouse line. Birth rates for all genotypes conformed to Mendelian ratios. In this mouse line, the internal ribosome entry site (IRES) allows co‐expression of the endogenous wild‐type *Otogl* allele and creERT2 under the control of the *Otogl* promoter. *Otogl* ^IREScre/+^ mice were gererated by crossing *Otogl* ^IREScreERT2/+^ mice with a flp deleter line to remove the F3‐ERT2‐F3 sequence,^[^ ^78^ ^]^ enabling co‐expression of the endogenous wild‐type *Otogl* allele and cre under the control of the *Otogl* promoter. Both male and female mice were used for experiments.

Genotyping of the *Bhlhb5* ^cre/+^ knock‐in recombinant, *Rosa*‐tdTomato Ai9 and *Otogl* conditional and knock‐out mouse lines had been described elsewhere.^[^ ^31^, ^55^, ^79^ ^]^ For *Otogl* ^List ofIREScre/+^ mice, genotyping was performed by Transnetyx, by real‐time PCR with the forward fOtogl‐3WT (5′‐GGAGGCTGCACGACAGT‐3′) and reverse rOtogl‐3WT (5’‐CATCACTGTTATTTAGGCACATGGC‐3’) oligonucleotides targeting the 3’UTR region of *Otogl*, and the forward fOtogl‐3KO (5’‐CCGATAGACTGCACATGCCAGT‐3’) oligonucleotide targeting exon 58 of *Otogl* and the reverse rOtogl‐3KO (5’‐CTAACGTTACTGGCCGAAGCC‐3’) oligonucleotide targeting the IREScreERT2 construct (Transnetyx, Cordova, TN).

### Sound Stimulation

Susceptibility to audiogenic seizures was assessed by delivering continuous pure tones from a calibrated loudspeaker (Vifa, Avisoft) placed on top of a large Perspex cylinder (30 cm tall, 15 cm diameter) containing the freely moving mouse. Audiogenic seizures typically occur as follows: The mouse enters a phase of wild running and then experiences tonico–clonic convulsions, tonic hyperextension of the hind limbs, and a potentially fatal postictal depression of consciousness. Sound stimulation was stopped as soon as the animal began running wildly, to prevent full‐blown seizures. We evaluated the proportion of mice displaying audiogenic seizures after stimulation with high‐intensity (100–110 dB) pure tones (10–14 kHz) lasting up to 1 min, during the P21–P28 and P35–P40 periods.

### Tissue Preparation

Heads of mouse embryos (before E12.5) were dissected in cold phosphate‐buffered saline (PBS) and fixed by immersion in 4% paraformaldehyde (PFA) in PBS for one day at 4 °C. Embryonic cochleae from mice (after E12.5) were dislodged from the skull in cold PBS and post‐fixed for 45 min in 4% PFA‐PBS. Tissues were then washed in PBS, protected by immersion in 30% sucrose, and embedded in optimal cutting temperature (OCT) compound (VWR International) for cryostat sectioning.

Adult mice were deeply anaesthetised with ketamine (200 mg kg^−1^) and xylazine (20 mg kg^−1^) by intraperitoneal injection before intracardiac perfusion with 4% PFA‐PBS and dissection. Each inner the ear was removed, the cochlea was extracted in cold PBS and its apex was perforated and fixed by incubation in 4% PFA‐PBS for 45 min at room temperature (RT). The organs were decalcified in 0.35 M EDTA (ethylenediaminetetra‐acetic acid) in PBS (pH 7.5) for one to two nights at 4 °C, washed in PBS and post‐fixed for 1 h in 4% PFA‐PBS. The cochleae were then protected by overnight immersion in 30% sucrose at 4 °C and embedded in OCT. Brains were fixed by incubation in 4% PFA‐PBS for 2 h at 4 °C, washed in PBS and protected by immersion in successive sucrose solutions, from 10% to 30% sucrose before embedding in OCT or in 2% agarose for free‐floating vibratome slicing (80–200 µm sections) with a Leica VT1000S (Leica Biosystems).

The resulting OCT blocks were frozen in Tissue Tek Cryomold Intermediate moulds (Q93740, Interchim) with liquid nitrogen or dry ice/isopentane, and were stored at −80 °C before cutting into 12 µm‐thick (cochlea), 20 µm‐thick (embryonic cochlea) or 60 µm‐thick (free‐floating brain sections from adults) cryostat sections (CryoStar NX70, Epredia). Sections were dried on Superfrost Plus slides (Menzel Gläser) for 1 h at RT and were then stored at −20 °C.

Whole‐mount cochleae were obtained by microdissecting cochleae under a dissection lens. Tissues were then processed on Superfrost slides (Menzel Gläser).

### Immunolabeling

Standard procedures were used to process samples for immunolabeling. The OCT was removed from the −20 °C storage slides of prepared tissues by washing with PBS, free‐floating tissues were directly processed after cryosection and in toto mounted cochleae were directly processed after extraction. The tissues were first permeabilised by incubation for 2 h at RT with 0.25% Triton X‐100 (Sigma‐Aldrich) in PBS. They were washed in PBS and blocked by incubation at RT for 2 h with 0.1% Triton X‐100 (Sigma‐Aldrich), 1% BSA (AppliChem), and 5% normal horse serum (NHS) in PBS for all tissues. Samples were then incubated with primary antibodies (**Table** **1**) in blocking solution overnight at 4 °C, washed in PBS and incubated with secondary antibodies for 2 h at RT in the dark.

**Table 1:** List of primary antibodies.

Primary antibodies (anti‐)	Host species	Dilution	Supplier	Reference
Calbindin (calb)	rabbit	1:1000	Cell Signaling	13176
Calretinin (CR)	rabbit	1:1000	Swant	7697
CtBP2	mouse	1:2000	BD Biosciences	612044
DARPP‐32	rabbit	1:1000	Abcam	ab40801
GATA3	goat	1:50	Bio‐Techne SAS	AF2605
GluA2	mouse	1:500	Millipore	MAB397
Alexa Fluor® 647 anti‐Myelin basic protein SMI‐99 (MBP)	mouse	1:500	BioLegend	808408
Myosin7a (Myo7a)	rabbit	1:100	Proteus	25‐6790
NF200	chicken	1:1000	Millipore	AB5539
Parvalbumin (PV)	mouse	1:1000	Swant	PV235
Pou4f1 (Brn‐3a)	mouse	1:50	Millipore	MAB1585
RFP	rabbit	1:1000	Bio Vision	3993‐100
Syt2 (Znp‐1)	mouse	1:1000	ZIRC	AB_10013783

For the labeling of ribbon synapses with anti‐GluA2, anti‐CtBP2, and anti‐Myosin7a antibodies, tissues were blocked directly by incubation for 1 h in 0.3% Triton X‐100, 20% NHS in PBS and were then incubated with primary antibodies overnight at 37 °C and then secondary antibodies for 2 h at 37 °C in 0.1% Triton X‐100, 1% NHS in PBS. For sectioned tissues stained with the anti‐Pou4f1 (Brn‐3a) antibody, antigen retrieval was performed before immunolabeling. Briefly, slides were placed on a platform above boiling water in a rice cooker and covered with sodium citrate buffer (10 mm sodium citrate, 0.05% Tween, pH 6.0). Slides were steamed in hot sodium citrate buffer for 5 min and were then incubated with RT sodium citrate buffer for 15 min. Slides were then washed three times in PBS and immunolabeled as described above.

The secondary antibodies listed in the following table were used (**Table** **2**). Nuclei were stained with DAPI (1:500, MBD0015; Sigma‐Aldrich) and samples were mounted on Superfrost slides (Epredia) with FluorSave Reagent (Calbiochem, Sigma‐Aldrich).

**Table 2:** List of secondary antibodies.

Secondary antibodies (anti‐IgG)	Host species	Conjugation	Dilution	Supplier	Reference
Chicken	Donkey	FITC	1:500	Thermofisher	SA1‐72000
Goat	Donkey	Alexa Fluor® 647	1:500	Abcam	ab150131
Mouse	Donkey	Alexa Fluor® 488	1:500	Abcam	ab150105
Mouse	Donkey	Alexa Fluor® 647	1:500	Abcam	ab150107
Rabbit	Donkey	Alexa Fluor® 488	1:500	Abcam	ab150073
Rabbit	Goat	Alexa Fluor® 555	1:1000	Abcam	ab150078
Rabbit	Donkey	Alexa Fluor® 647	1:500	Abcam	ab150075

For quantifications of type IA, IB, and IC neurons as well as the distribution of tdTomato‐positive neurons at the apex, middle, and base of the cochlea (Figures 3, 4, and 8; Figure S9,S11, Supporting Information), cochlear sections from both cochleae of 3 animals were analysed for each genotype.

### RNA in Situ Hybridization and Immunohistofluorescence Assays

RNA in situ hybridization assays were performed with the RNAscope kit (RNAscope Multiplex Fluorescent Reagent Kit v2 Assay, ACD, catalogue no. 323100) from Advanced Cell Diagnostics (Bio‐Techne SAS, Rennes), according to the manufacturer's instructions. The OCT was removed in PBS, slices were heated for 30 min at 40 °C and post‐fixed by incubation in 4% PFA for 15 min at RT. The slides were then washed in H_2_O, dehydrated in a series of ethanol solutions, and dried for 30 min at 40 °C before incubation with hydrogen peroxide for 10 min at RT to saturate endogenous peroxidases and heating in a water bath oven for 5 min at 99 °C in antigen‐unmasking buffer. Tissues were then incubated in absolute ethanol, digested with Protease III solution in a hybridization oven at 40 °C for 30 min and hybridized with the target probes at 40 °C for 2 h. Target RNAscope Probe‐Mm‐Otogl‐C1 (Cat. No. 567271, ACDBio‐Techne), RNAscope Probe‐Mm‐Neurod1‐C2 (Cat. No. 416871‐C2, ACDBio‐Techne) and RNAscope Probe‐Mm‐Prph‐C2 (Cat. No. 400361‐C2, ACDBio‐Techne) contained a mixture of short oligonucleotides binding to a specific target mRNA and detectable through their C1 or C2 channel tags. The Opal520 and/or Opal650 fluorophore dyes (Akoya Biosciences, Menlo Park, USA) were used to reveal the RNAscope C1/C2 channel tags. The tissue was washed several times and subjected to sequential hybridization procedures with the RNAscope Multiplex Fl V2 Amp1, Amp2 and Amp3, and horseradish peroxidase, to amplify the signal. The fluorescent probes Opal520 and/or Opal650 (1/1000 dilution) were then added for channel C1 and/or C2 labeling. When tdTomato protein detection was required, additional immunofluorescence staining was performed after the RNAscope procedure. The tissues were incubated in a blocking solution (0.1% Triton‐X100, 1% BSA, 5% NHS) for 1 h at RT, and then with the primary antibody overnight at 4 °C, followed by the secondary antibody for 2 h in the dark at RT. The antibodies used were a rabbit primary antibody directed against RFP (1/1000, Bio Vision 3993‐100) and a goat anti‐rabbit Alexa fluor 555 (1/500, Abcam ab150078) conjugated secondary antibody. DAPI (MBD0015; Sigma‐Aldrich) was then added at a dilution of 1/500, for 10 min at RT. The slides were mounted in FluorSave Reagent (Calbiochem, Sigma‐Aldrich).

### Transmission Electron Microscopy

Cochleae from 4 *Otogl* ^+/+^ and 4 *Otogl* ^+/−^ mice were fixed in 2% paraformaldehyde and 2.5% glutaraldehyde in 0.1M PBS (pH = 7.4) for 2 h at RT. P23–P25 cochleae were washed in PBS and decalcified in 0.35M EDTA‐PBS for 48 h. All samples were then washed in water, postfixed in osmium tetroxide for 1 h, washed in water, and dehydrated in acetone series from 70% to 100%. Samples were embedded in Spurr resin (EMS), followed by polymerization for 48 h at 60 ° C. Ultrathin sections (70 nm) were cut with a Leica Ultracut UC7 microtome, stained with uranyl acetate and then lead citrate. Transmission electron microscopy images of one cochlea for each animal were captured with a Tecnai Spirit 120 kV TEM equipped with a bottom‐mounted Eagle 4k × 4k camera (FEI, USA). The g‐ratio, the ratio between the inner axonal diameter and the total neuron diameter, was measured along the shortest axis of the neuronal sections. To minimize localization bias, several regions were imaged between IHCs and the spiral ganglion for each animal and were then processed (see distribution in Figure 6C). A minimum of 200 neurons on 4–8 images were counted for each animal. To further minimize localization bias, average values of the axon diameter and g‐ratio were extracted for each image.

### Fluorescence Microscopy

Images were acquired with an LSM‐900 Airyscan confocal microscope (Zeiss, Oberkochen, Germany) or a Nikon Eclipse Ti2 microscope (Nikon, USA), with laser excitation at 405, 560, and 640 nm. Plan Apochromat 1.4 numerical aperture (N.A.) 63× oil immersion, Apochromat 1.1 NA 40× oil immersion, Plan Apochromat 0.8 NA 20× air, and Plan Neofluar 0.16 NA 5× air objectives were used for the Zeiss microscope and Plan Apochromat Lambda S 1.25 NA 40× silicone immersion, Plan Apochromat 1.05 NA 25× silicone immersion, and Plan Apochromat Lambda 0.45 NA 10× air objectives were used for the Nikon microscope. Images were Z‐projected, adjusted for brightness and contrast, converted to RGB format with Fiji‐ImageJ (NIH) and processed with Photoshop (Adobe) softwares.

### Functional Hearing Tests

Mice were anaesthetised by intraperitoneal injection of a mixture of ketamine (150 mg kg^−1^, Virbac, France) and xylazine (4.5 mg kg^−1^, Rompun, France). Body temperature was maintained at 37 °C (Microprobe Thermometer, BAT‐12, WPI, UK) throughout all procedures, with a warming pad coupled to a rectal probe (Homeothermic Blanket System, Harvard Apparatus).

Auditory brainstem responses (ABRs)^[^ ^80^ ^]^ were recorded in response to pure tone pips at frequencies of 2 to 32 kHz and at sound levels of 15 to 105 dB sound pressure level (dB SPL). ABRs were averaged over 100–200 tone pip stimulus presentations. ABR thresholds were defined as the lowest stimulus level resulting in recognisable waves. ABR wave I amplitude was estimated by measuring the voltage difference between the wave I peak and the trough between waves I and II, and ABR wave I latency was measured as the time from sound stimulation to the wave I peak. ABR wave V amplitude was estimated by measuring the voltage difference between the wave V peak and the subsequent trough. Waves I and V amplitudes were measured for sounds at 75, 85, 95, and 105 dB SPL. Electrode responses were amplified (gain of 10 000), filtered, digitally converted, and averaged with a CED 1401+ data acquisition system (Cambridge Electronic Design Limited, Cambridge).

DPOAEs were collected with a miniature microphone at the entry to the auditory canal.^[^ ^81^ ^]^ Two primary pure tone stimuli of frequencies *f1* and *f2* were applied simultaneously. Frequency *f2* was set to various values between 5 and 20 kHz and the *f2/f1* ratio was kept constant at 1.2. The cubic difference tone at *2f1*−*f2*, the most prominent distortion product generated by mammalian ears, was measured for primary tone frequencies of equal intensity, from 40–75 dB SPL.

A two‐step masking ABR protocol was used to assess the response of low SR neurons. ABRs were first measured at 75 dB SPL to record high SR neuron activity. Continuous broadband Gaussian noise was then produced by a wave generator (8904A Multifunction Synthesizer DC–600 kHz, Hewlett‐Packard) and attenuated, if necessary, with an attenuator (PA5, Tucker‐Davis Technologies) before delivery to the auditory canal via a calibrated earphone (FT17 with closed‐field adapter, Fostex, Japan). This masker had a flat spectrum (± 5 dB) between 3 and 50 kHz. Masking was gradually increased by 1 dB steps until the ABR to the 75 dB SPL 10 kHz tone pip was erased. Masking level was then kept constant for the rest of the experiment. For complete masking of the ABR, the masker had to saturate the contributing high SR neurons, probably most of the medium SR neurons, and, due to its broad spectrum, it probably also saturated high SR neurons along most of the cochlear spiral. The probe tone used to generate the ABRs was then increased by 10 dB steps. This complete protocol generated a reABR signal in five waves, reflecting the activity of SGNs with thresholds sufficiently high to prevent saturation by the masker,^[^ ^44^ ^]^ corresponding to low SR neurons, which were recruited by this increase in sound intensity from 85 to 105 dB SPL in 10 dB steps. For the comparison of reABR signals between genotypes, the amplitude of the reABR waves was normalised by calculating ratio of reABR and ABR amplitudes for a given wave. ReABR assessment required a dynamic range for hearing of ≈ 80 dB, precluding its use in *Otogl* ^−/−^ mice, which had a hearing loss of at least ≈ 60 dB across all frequencies.^[^ ^31^ ^]^

### Electrophysiological Patch‐Clamp Recordings

Electrophysiological patch‐clamp recordings were performed on excised cochlear apical coils from P25–P35 *Otogl* ^+/+^, *Otogl* ^+/−^ and *Otogl* ^−/−^ mice, as previously described.^[^ ^53^, ^82^ ^]^ The dissection solution contained 143 mm NaCl, 6 mm KCl, 1.3 mm CaCl_2_, 0.9 mm MgCl_2_, 0.7 mm NaH_2_PO_4_, 5 mm glucose, 2 mm sodium pyruvate, and 10 mm HEPES with a pH of 7.4. Recordings were performed at RT (20–23 °C). The patch pipette electrodes were made of borosilicate glass (World Precision Instruments).

For cell capacitance measurements on IHCs, the pipette resistance in the external solution was 2–3 MΩ. Only cells with a series resistance below 10 MΩ (uncompensated) were included in the study. Ca^2+^ current and ΔC_m_ were recorded with an EPC‐9 patch‐clamp amplifier and Patchmaster software (HEKA, Ludwigshafen, Germany). We used a single 30 mV amplitude sine wave from a holding potential of −95 mV. The acquisition frequency was 50 kHz, low‐pass filtered at 6–10 kHz, and the frequency of the sine wave was 1 kHz. The resulting maximal depolarization to ≈ −65 mV was sufficiently small to avoid Ca^2+^ current activation. Ca^2+^ current recordings were corrected for the linear leak conductance measured near −95 mV. Liquid junction potential (≈ −15.5 mV) was corrected off‐line for Cs–gluconate based intracellular solutions. The extracellular recording solution consisted of 111.5 mm NaCl, 6 mm KCl, 10 mm CaCl_2_, 1 mm MgCl_2_, 27 mm TEA‐Cl, 2 mm sodium pyruvate, 5 mm glucose, 10 mm Na‐HEPES with a pH of 7.4 (adjusted with NaOH). Tetrodotoxin (1 µm) and apamin (1 µm) were added to the extracellular solution. The intracellular pipette solution for cell membrane capacitance recordings contained 140 mm Cs–gluconate, 20 mm TEA‐Cl, 0.5 mm EGTA, 5 mm creatine phosphate, 4 mm Mg‐ATP, 0.3 mm Na_2_‐GTP, 10 mm HEPES with a pH 7.2 of (adjusted with CsOH). The ΔC_m_ evoked by membrane depolarisation was measured as ΔC_m_ = C_m (response)_ – C_m (baseline)_ and was used to estimate synaptic vesicle exocytosis in IHCs. C_m (baseline)_ was obtained by averaging capacitance data points before the depolarising pulse and C_m (response)_ was obtained by averaging capacitance data points after the transient current following the depolarising pulse.^[^ ^53^ ^]^

### Audiogenic Seizure in Vivo Electrophysiological Recordings

Ten animals (7 *Otogl* ^−/−^ mice, 3 *Otogl* ^+/+^ mice, P26–P55) were used for the recording of brain activity during the elicitation of an audiogenic seizure (Figure 1B,C). Each animal underwent a 1.5 h surgical procedure with sterilized instruments. The animal was first sedated by subcutaneous injection of a mixture of medetomidine (0.1 mg kg^−1^) and buprenorphine (0.1 mg kg^−1^), and then anaesthetized with isoflurane (4% for induction and then 2%, with 95% O_2_). The animal's head was shaved, and an ophthalmic lubricant was applied to the eyes. Lidocaine (10 mg kg^−1^) was injected subcutaneously directly above the skull. The skin was carefully cleaned with betadine and ethanol. A clean incision was made on the skull, large enough for the placement of three pins (two for recording and one for reference) and a holding head post. The excess skin was removed, and skin edges were glued to the skull with surgical glue. The vasodilator properties of lidocaine made it possible to observe the vessels under the skull and, thus, locate the areas of interest: the left auditory cortex and the left inferior colliculus. A hole was drilled above the auditory cortex with a compact milling machine (burr diameter 1.2–1.5 mm). A 0.6 mm pin (tin, 8 mm long, from a classical male pin header connector, RS Components, Corby, United Kingdom) was inserted into the hole in contact with the dura and was glued to the skull. The same method was applied for the pin above the inferior colliculus and the reference pin was positioned above the right frontal area. Finally, the headpost was fixed to the skull with dental cement (Figure 1B). Meloxicam (Metacam, 5 mg kg^−1^) was injected subcutaneously post‐surgery before the animals awoke, and then once daily (1 mg mL^−1^) for three days. In case of infection, sulfadoxine‐trimethoprim was injected subcutaneously (Borgal, 20 mg kg^−1^). The weight of the animals had typically increased or had stabilized by third day post‐surgery. Buprenorphine injections (1 mg kg^−1^) were administered subcutaneously, as needed, based on pain score evaluation. Animals were acclimated to head fixation for a few days before the experiments.

For recordings, the animals were placed in a stereotaxic frame with the headpost fixed to the frame (Figure 1B) in an acoustically and electromagnetically isolated chamber. The epidural pins of the animal were connected to a TDT RA4 preamplifier and a Tucker‐Davis Technologies (TDT, Alachua, USA) RZ6 recording station. The RZ6 also transmitted the sounds to a free‐field (TDT ES1) transducer. Electrophysiological responses were recorded from epidural pins at a sampling rate of 5 kHz. Signals were subjected to a Butterworth filter between 3 Hz and 3 kHz and were displayed and analyzed with Matlab 2022a (Mathworks). For EEG, spontaneous neural activity was recorded from the inferior colliculus and auditory cortex for ≈30 s before a pure tone at 10 kHz (or 14 kHz if no crisis occurred at 10 kHz) was presented at 105 dB SPL. Animals were filmed during recordings (Cineplex, Plexon Inc., USA). The sound was stopped 3 to 5 s after the onset of signs of an audiogenic seizure (clonic phase), and recording was stopped after ≈1 min. The state of the animal was carefully checked and it was reintroduced into its home cage. The tone‐to‐baseline ratio was calculated by carrying out the ratio of the spectrum during tone presentation to that in the 20 s before the tone. For ripple detection, the EEG signal was bandpass‐filtered in the 120 – 600 Hz range. A ripple was considered to occur when the temporal envelope of the filtered EEG signal exceeded three standard deviations above the baseline signal in the absence of a ripple.

### Inferior Colliculus In Vivo Electrophysiological Recordings

Seventeen animals (8 *Otogl* ^+/+^, 9 *Otogl* ^+/−^, P29–P40) for microelectrode recordings were used. The animals were placed in standard housing in 12 h light/12 h dark conditions, with a background noise of < 40 dB SPL. They had unlimited access to food and water and were housed in cages with a maximum of five mice each.

Surgery (craniotomy around the lambda mark) was performed under ketamine (190 mg kg^−1^) and xylazine (4.5 mg kg^−1^) anesthesia. Recordings were performed under isoflurane (0.8% to 1% isoflurane at a flow rate of 0.2 L min^−1^ of 95% O_2_) anesthesia 1 h after the intraperitoneal injection. A 32‐channel laminar electrode (NeuroNexus, Ann Arbor, USA) was implanted in the central nucleus of the inferior colliculus (visible under the skull), typically at a depth between 500 and 1800 µm. The high‐frequency signal (0.3–10 kHz) acquired by the electrophysiology system (OmniPlex, Plexon Inc., USA) included the extracellular action potentials of neurons. STRFs of inferior colliculus neurons in response to a 50 ms pure tone were measured as firing rate as a function of tone frequency and time. The neural response was characterized by extracting the bandwidth of the significant response area, the latency of the neural response, the maximum evoked firing rate, and the baseline activity level (average firing rate in the 10 ms preceding the tone stimulation) from the STRF (Figure 9B,D). Responses to 30 repeated click trains (click rates between 2 Hz and 480 Hz) were also recorded. Clicks were 100 µs long. The ASSR was defined as the average response to each click (Figure 9E). The ASSR maximum firing rate relative to that of the first click (onset response) was analyzed as a function of click rate to measure the progressive decrease in phase‐locking ability with increasing click rate. During a recording session, the laminar electrode was typically implanted five to seven times. Animals were then killed by cervical dislocation.

### Startle Reflex

The acoustic startle reflex caused a rapid stiffening of the limbs, body wall, and dorsal neck following the presentation of a sound with an intensity over 80 dB SPL.^[^ ^83^ ^]^ Acoustic startle reflex measurements were performed in *Otogl* ^+/+^, *Otogl* ^+/−^, and *Otogl* ^−/−^ mice between P20 and P25 (*n* = 10), on three successive days, according to the SR‐LAB System procedure (6310‐0000‐U, San Diego, CA). Each animal was placed in the startle enclosure of an isolation cabinet associated with a free‐field calibrated speaker and movements were recorded with a piezoelectric ultra‐sensor base plate positioned beneath the animal. The electrical currents recorded following the movement of the animal were amplified and processed with SR‐LAB software. The standard startle reflex response with random sound intensities from 65 to 110 dB SPL was initially measured, in 5 dB steps. In total, 11 trials with 119 repetitions were performed in the presence of continuous background noise at 45 dB SPL, corresponding to the ambient noise of the experimental room, and startle responses were recorded over a period of 150 ms. The first startle stimulus was presented after 5 min of acclimatization, and the others were separated by a random intertrial interval (ITI) of 10–19 s. In the second part of the protocol, acoustic stimuli were embedded in a continuous background of 70 dB SPL with the same trial and repetition conditions as for the first part of the protocol. Gap inhibition of the startle reflex was tested in the presence of 70 dB SPL background noise at 110 dB SPL, with a 50 ms interval between the end of the gap and the onset of the 50 ms stimulus. Data were then centralized with SR‐LAB analysis software to record the onset, amplitude, and latency of the stronger response, for which a reaction time of 15–70 ms was considered as a reflex for a startle amplitude greater than 5 mV.

### MEMR

DPOAEs generated by a bitonal sound stimulus at frequencies *f1* and *f2* can be used to study the MEMR, as their frequency, *2f1−f2*, can be fixed to optimize detection of the MEMR. The stimulus for DPOAE measurements was two tones at *f2* = 9 kHz and *f1* = 7.5 kHz (i.e. *2f1−f2* = 6 kHz) sent to the right the ear at an intensity of 50 dB SPL (below MEMR threshold) with fixed‐onset phases. The *2f1−f2* distortion product was collected at the right ear. The MEMR‐inducing sound was presented to the left the ear via a calibrated earphone placed in the auditory canal, to prevent acoustic interference with DPOAE detection. This MEMR‐inducing sound was a band pass‐filtered white noise [0.02–5 kHz] designed to have a maximal triggering efficiency and to generate minimal cross‐talk with DPOAE recordings in the contralateral ear, increasing from 40 to 90 dB SPL in 5 dB steps. When the noise intensity was sufficient to induce a mean DPOAE level and phase shift (*2f1−f2*), this intensity was considered to be the threshold for MEMR activation.^[^ ^23^ ^]^ *Otogl* ^−/−^ mice had no DPOAE due to the uncoupling of OHCs from the tectorial membrane. Therefore, it was not possible to probe the stapedial reflex in these mice.

### Statistics and Data Analysis

All statistical analyses were performed with Igor Pro (WaveMetrics, Portland, OR, USA), Prism (Graphpad, La Jolla, CA, USA), and Matlab 2022a (Mathworks, CA, USA) softwares. The following tests were performed, as appropriate, according to the data: Fisher's exact test to compare proportions (Figures 1, 4, and 8), Student's *t*‐test (Figure 1Ci), Student's *t*‐test between two means with Welch's correction which does not assume equal variances (Figures 1, 3, 4, 5, 6, 7, 8, 10 and Figure S9,S10, Supporting Information), Mann‐Whitney's test (Figure 10B) when the data could not be assumed to be normally distributed, two‐sample Kolmogorov‐Smirnov test to compare distributions (Figure 6C) and two‐way ANOVA (Figures 7, 9, and 10A). For electrophysiological in vivo data (Figure 9), as multiple neurons were recorded within each animal, we used nested two‐way ANOVA (Figure 9Di,ii) or three‐way ANOVA (Figure 9E) with the animal factor nested into the group factor.

Statistical significance with the associated test was indicated throughout the text and in the figures as follows: ns, not significant; ^*^ *p* < 0.05; ^**^ *p* < 0.01; ^***^ *p* < 0.001, except in Figure 1Ci where significance (*p* < 0.05) was indicated with a horizontal dashed bar for readability. All values were given as means ± standard deviation. The number of biological replicates and/or animals used was indicated in brackets within the figures.

## Conflict of Interest

C.P. and P.A. are members of the scientific advisory board of Sensorion.

## Author Contributions

B.G., P.A., and N.M. are joint senior authors. N.M. conceived the project. N.M., P.A., and B.G. co‐supervised the work. M.G., S.M., and C.C.P contributed equally to this work and co‐performed most of the experimental work. M.G., S.M., A.S.E., S.U., T.C.I., M.M., and E.V. carried out postnatal histological work. S.M., C.M.B.S., and R.E. carried out embryonic histological work. T.D. managed mouse lines. N.M. and B.L.P. initiated studies on *Otogl*. P.A. carried out ABR and reABR recordings. F.G. carried out MEMR measurements. J.S. and B.B. analysed cochlear nucleus circuits. M.G., P.J., S.D., and A.E.A. carried out ribbon synapse experiments. C.S. and A.M. carried out and conducted the transmission electronic microscopy work, respectively. C.C.P., N.M., T.D., O.P., and B.G carried out patch‐clamp and in vivo electrophysiology work. M.G. and S.M. processed the raw data and conducted the histological data analysis with the help of N.M., P.A., F.G., B.G., and N.M. processed the raw data and conducted the electrophysiological data analysis. N.M., S.M., M.G., P.A., C.P., and B.G. wrote the paper. All the authors read and approved the manuscript before submission.

## Supporting information



Supporting Information

## Data Availability

All data needed to evaluate the conclusions in the paper are present in the paper and/or the Supplementary Material. The data can be provided by the corresponding author pending scientific review and a completed material transfer agreement. Requests for the data should be submitted to N. Michalski.
