# Supplementary material for: Deciphering Auditory Hyperexcitability in Otogl Mutant Mice Unravels an Auditory Neuropathy Mechanism
Source: Adv Sci (Weinh). 2025 Feb 18;12(19):2410776. doi: 10.1002/advs.202410776 (PMC12097039; doi:10.1002/advs.202410776)
Supplement: Supplementary file 1 — Supporting Information [file ADVS-12-2410776-s001.docx]

**Supporting Information**

**Deciphering Auditory Hyperexcitability in *Otogl* Mutant Mice Unravels an Auditory Neuropathy Mechanism**

Mathilde Gagliardini^1,2^†, Sabrina Mechaussier^1^†, Carolina Campos Pina^1,2^†, Monica Morais^1^, Olivier Postal^1,2^, Philippe Jean^1^, Typhaine Dupont^1^, Amrit Singh-Estivalet^3^, Shéhanie Udugampolage^1^, Cyril Scandola^4^, Elisabeth Verpy^5^, Baptiste Libé-Philippot^1^, Talya C. Inbar^1^, Joanna Schwenkgrub^6^, Carla Maria Barbosa Spinola^7^, Raphaël Etournay^7^, Aziz El-Amraoui^8^, Brice Bathellier^6^, Adeline Mallet^4^, Sedigheh Delmaghani^8^, Fabrice Giraudet^9,10^, Christine Petit^3,11^, Boris Gourévitch^1^‡, Paul Avan^9,12^‡, Nicolas Michalski^1^‡^*^


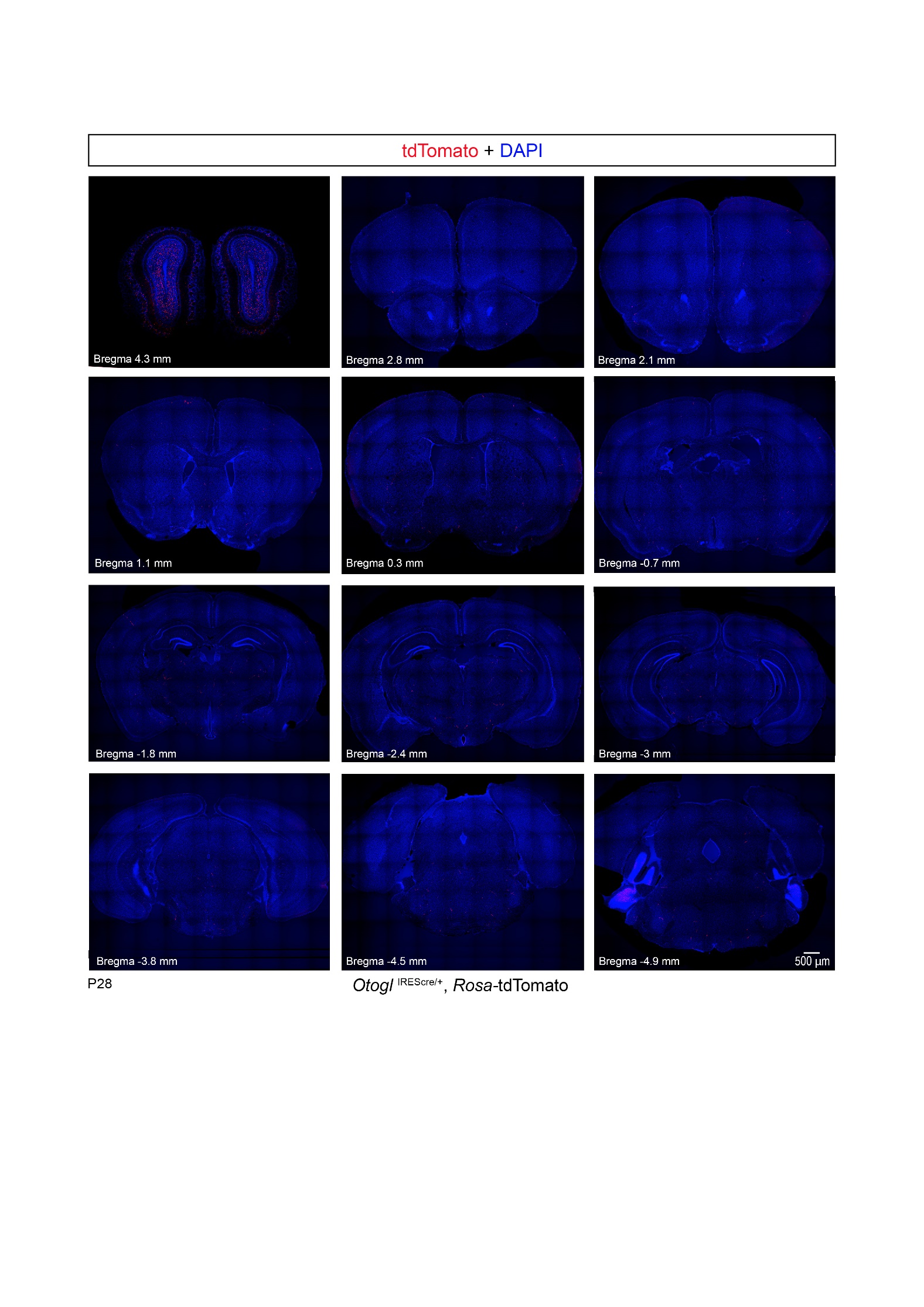


**Figure S1. Small numbers of tdTomato-positive cells in the brains of *Otogl*^IREScre/+^:*Rosa-*tdTomato mice**. Coronal sections along the postero-anterior axis (from top left to bottom right) of the brain of a P28 *Otogl*^IREScre/^:*Rosa-*tdTomato mouse. The approximate position of the slice relative to Bregma is indicated. Cell nuclei are stained in blue (DAPI).

**
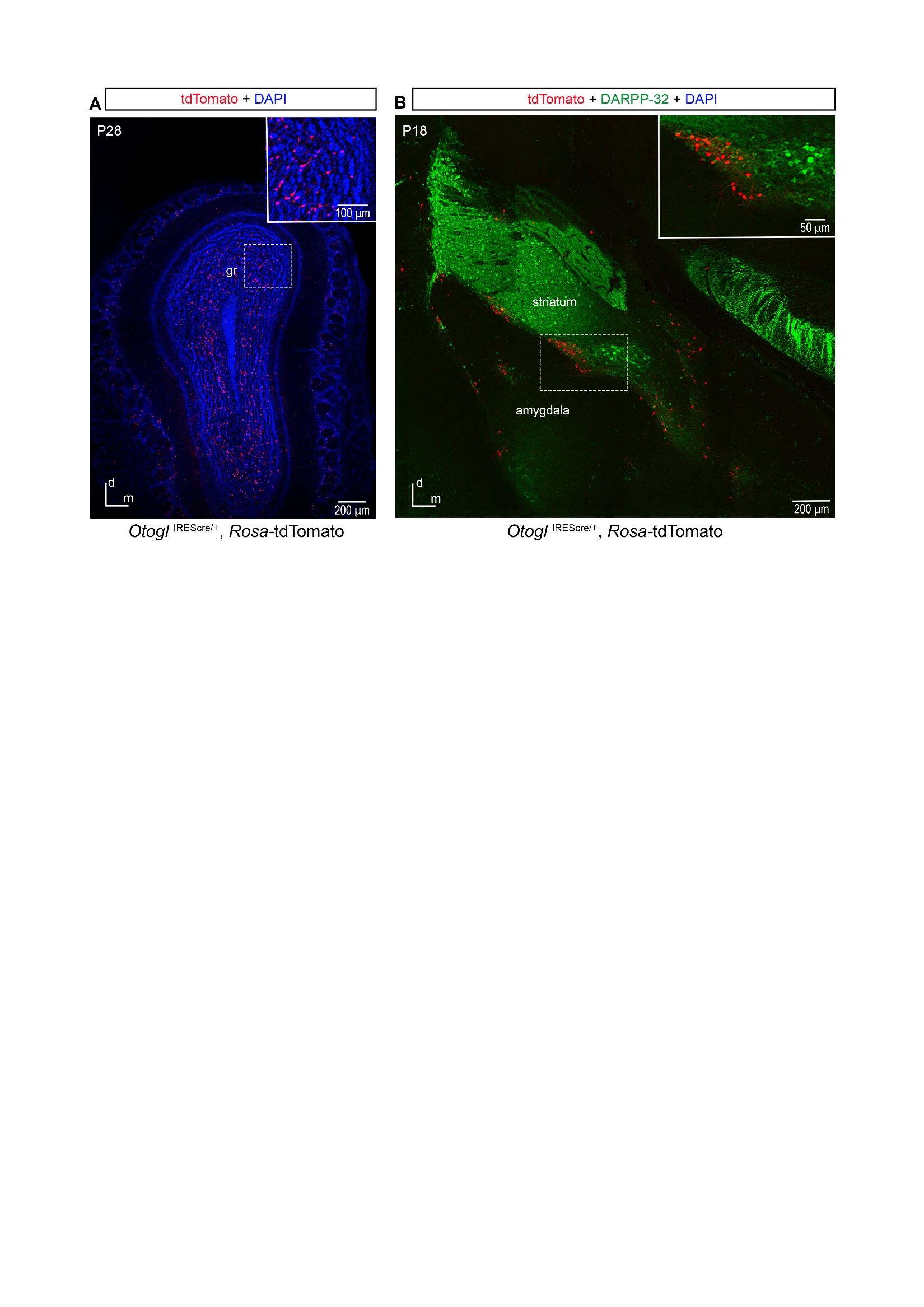
**

**Figure S2. TdTomato-positive cells are present in the olfactory bulb and peristriatal regions** **of *Otogl*^IREScre/+^:*Rosa-*tdTomato mice**. **A**) Coronal section of the olfactory bulb of a P28 *Otogl*^IREScre/+^:*Rosa-*tdTomato mouse in which some granular neurons are tdTomato-positive. **B**) Coronal section of the striatal region of a P18 *Otogl*^IREScre/+^:*Rosa-*tdTomato mouse immunostained for the GABAergic striatal medium spiny neuron marker DARPP-32 (green)*.* Cell nuclei are stained in blue (DAPI). d, dorsal; gr, granular layer; m, medial.

**
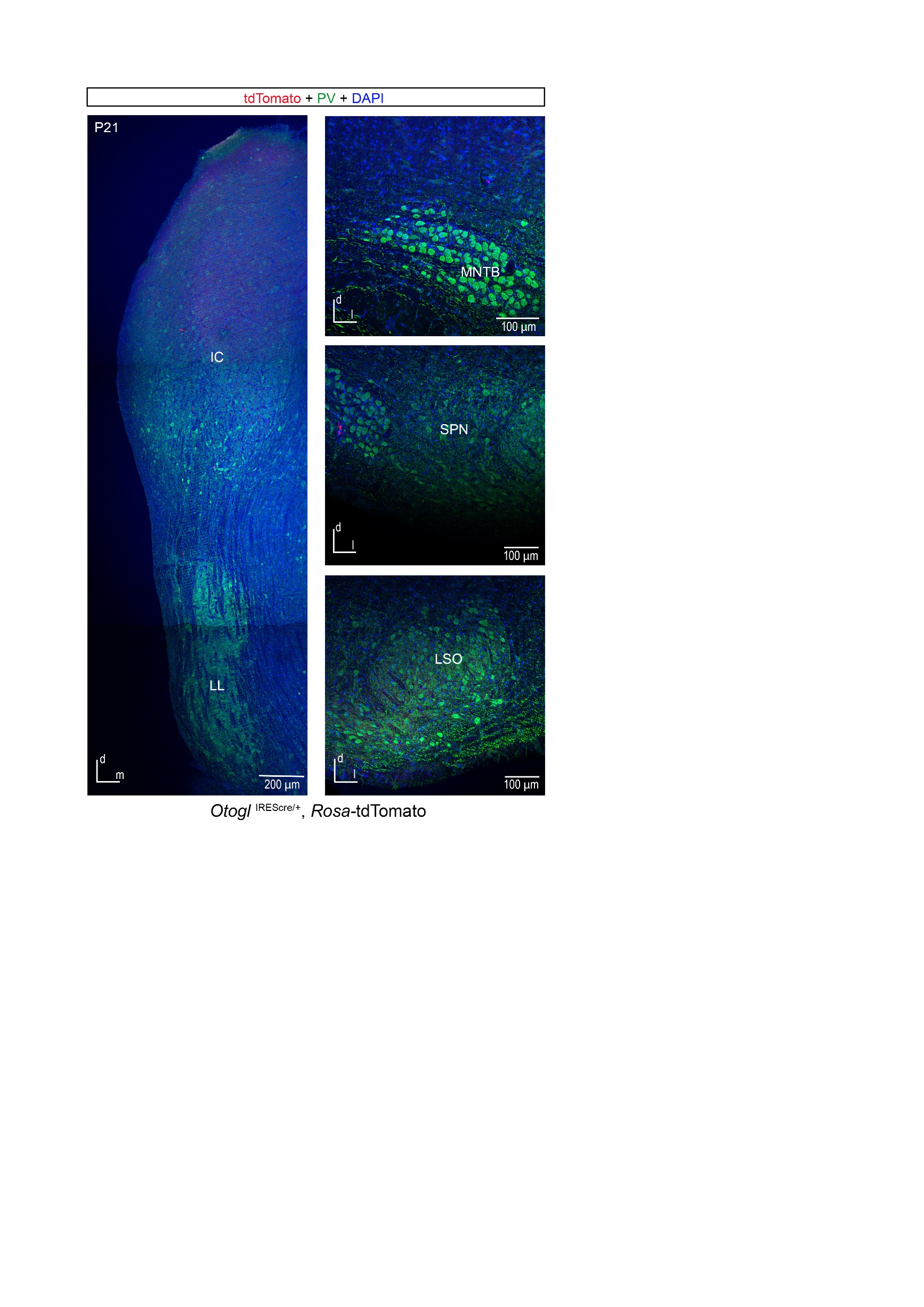
**

**Figure S3. Absence of tdTomato-positive cell in the superior olive and the auditory midbrain of *Otogl*^IREScre/+^:*Rosa-*tdTomato mice**. Coronal sections of the auditory midbrain (left) and superior olive (right) of a P21 *Otogl*^IREScre/+^:*Rosa-*tdTomato mouse immunostained for parvalbumin (PV) (green), a molecular marker of auditory brainstem neurons. Cell nuclei are stained in blue (DAPI). d, dorsal; IC, inferior colliculus; l, lateral; LL, lateral lemniscus; LSO, lateral superior olive; m, medial; MNTB, medial nucleus of the trapezoid body; SPN, superior periolivary nucleus.


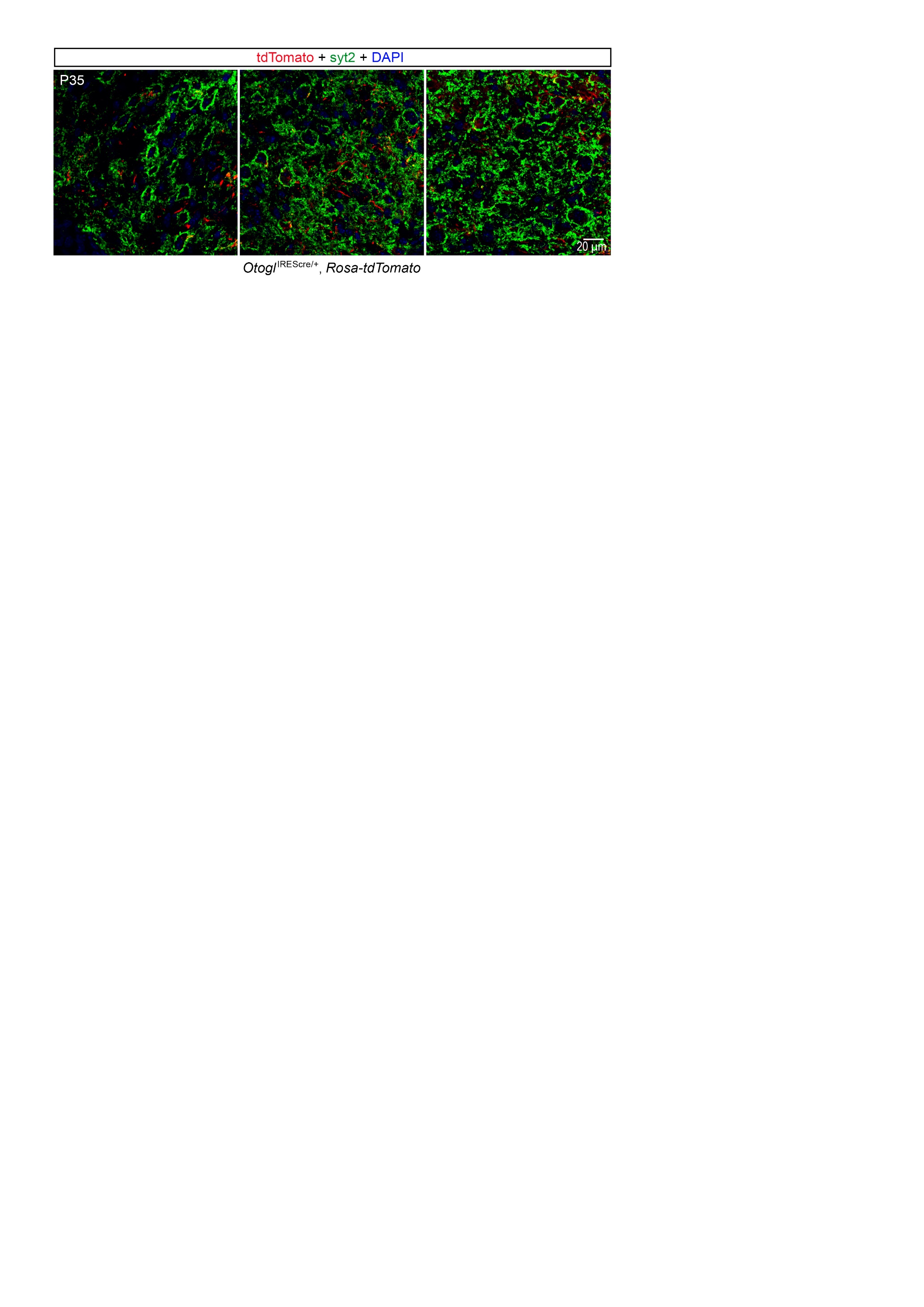


**Figure S4. Absence of tdTomato-postive endbulbs of Held in *Otogl*^IREScre/+^:*Rosa-*tdTomato mice.** Coronal sections of the CN from three P35 *Otogl*^IREScre/+^:*Rosa-*tdTomato mice immunostained for the endbulb of Held synaptic marker synaptotagmin-2 (green). Cell nuclei are stained in blue (DAPI).


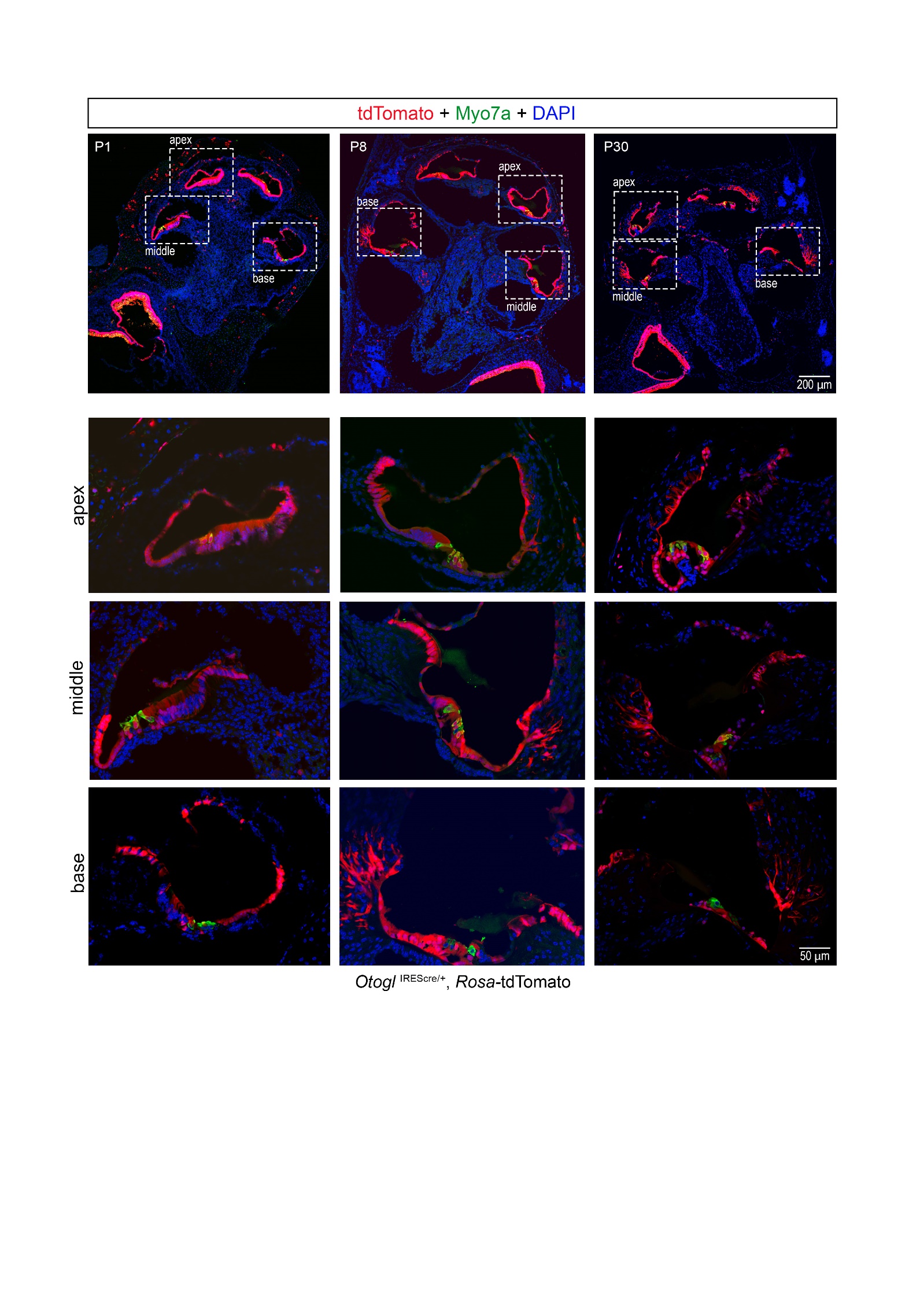


**Figure S5. TdTomato-positive cells in the cochlea of *Otogl*^IREScre/+^:*Rosa-*tdTomato mice.** Longitudinal cross-sections of the cochlea of *Otogl*^IREScre/+^:*Rosa-*tdTomato mice on P1 (top left), P8 (top middle) and P30 (top right), with immunostaining for Myosin7a (Myo7a) (green) and enlarged views of the insets showing the apical, middle and basal cochlear canals. Of note, the brightness of the red channel was increased in overview images of the cohleae (top row) to distinguish tdTomato labelling both in the organ of Corti and in SGNs. Cell nuclei are stained in blue (DAPI).


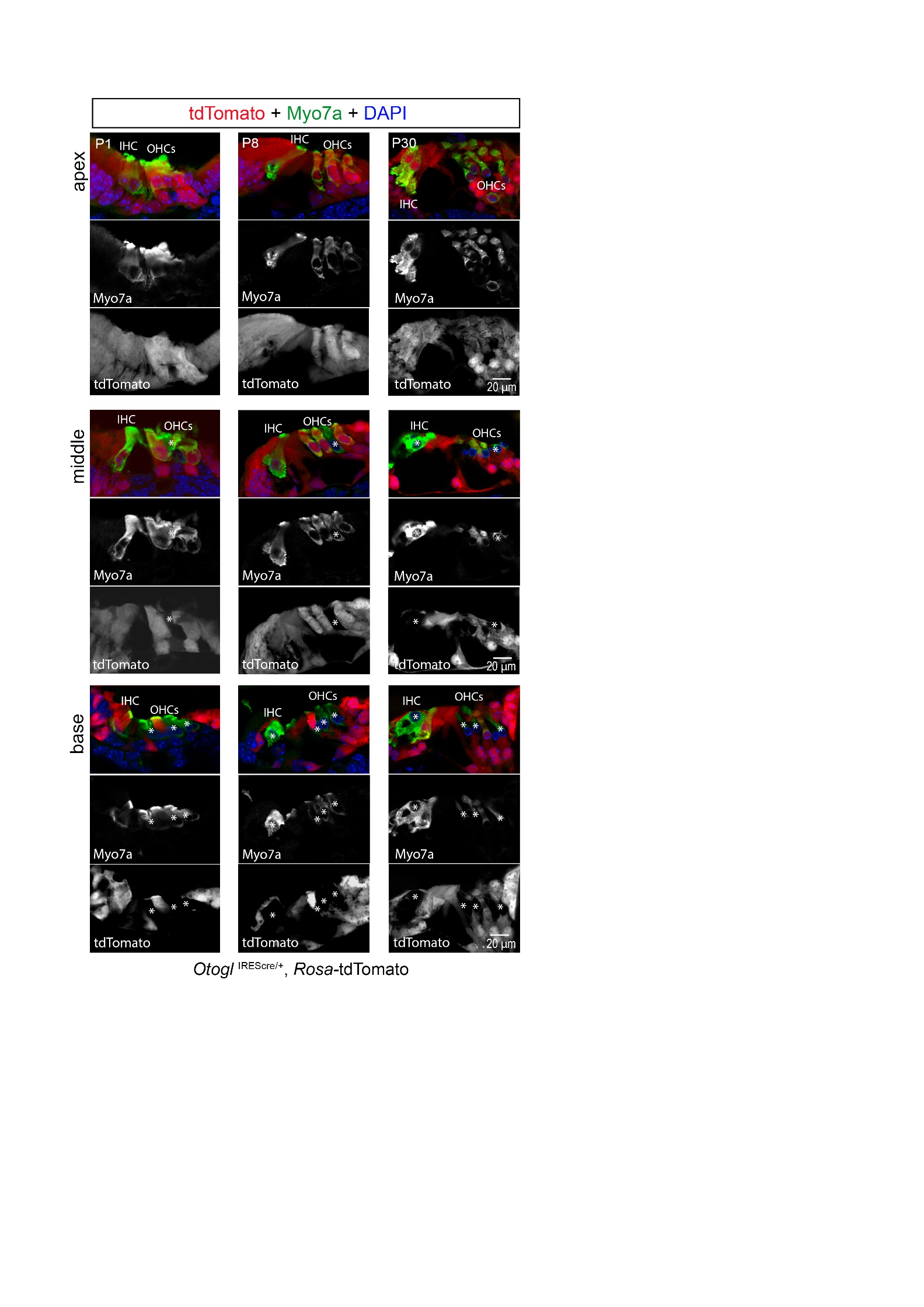


**Figure S6.** **TdTomato-positive cells in the organ of Corti of *Otogl*^IREScre/+^:*Rosa-*tdTomato mice.** Longitudinal cross-sections of the organs of Corti of *Otogl*^IREScre/+^:*Rosa-*tdTomato mice on P1 (left), P8 (middle) and P30 (right), with immunostaining for Myosin7a (Myo7a) (green) at the apex (top), middle (middle) and base (bottom) of the cochlea. Cell nuclei are stained in blue (DAPI). Asterisks mark the positions of some tdTomato-negative hair cells. Of note, tdTomato was not detected in the IHCs and OHCs of the basal region of cochlea and in some hair cells of the middle region. IHC, inner hair cell; OHCs, outer hair cells.


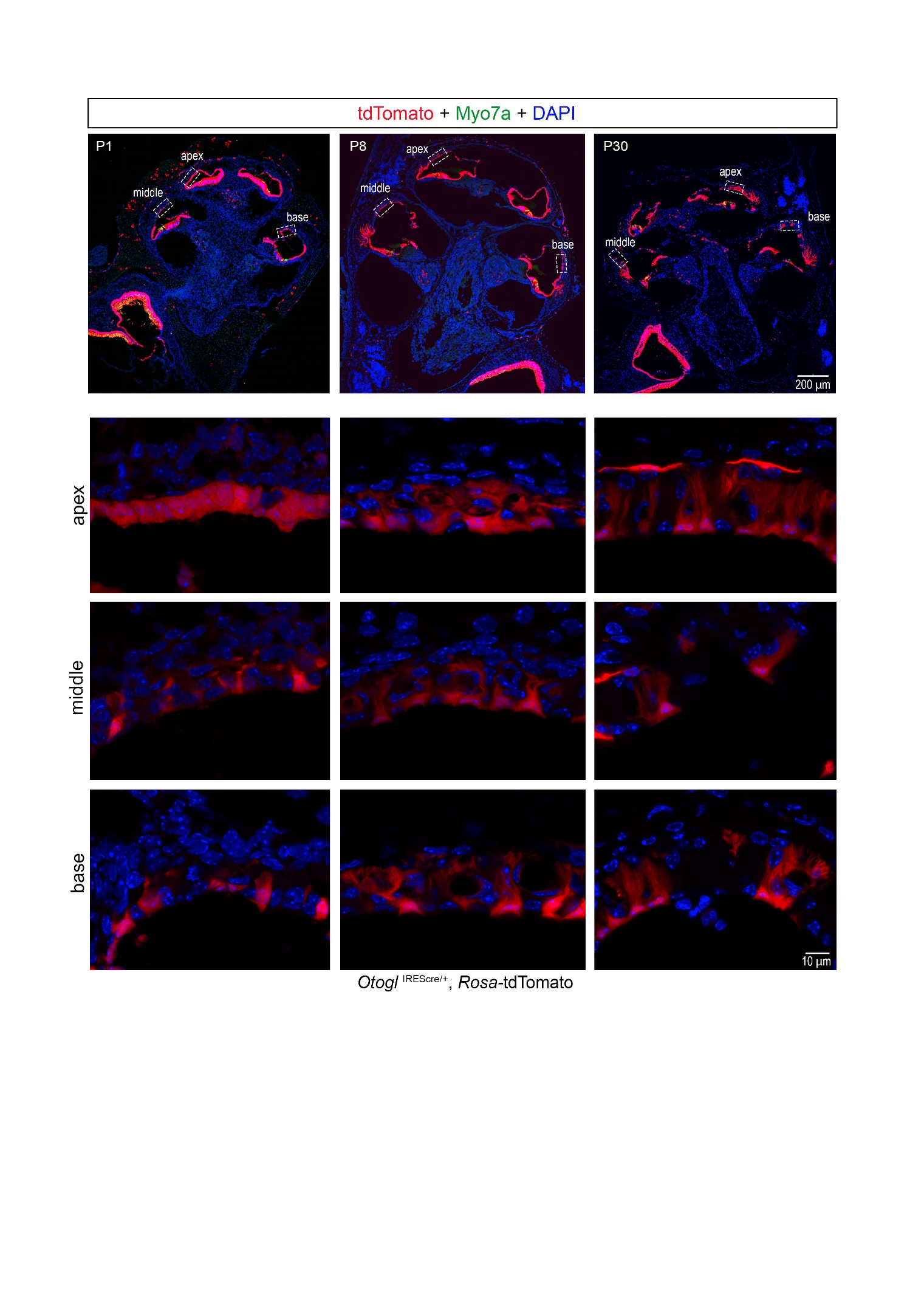


**Figure S7. TdTomato-positive cells in the stria vascularis of *Otogl*^IREScre/+^:*Rosa-*tdTomato mice**. Longitudinal cross-sections of the cochleae of *Otogl*^IREScre/+^:*Rosa-*tdTomato mice on P1 (top left), P8 (top middle) and P30 (top right), with immunostaining for Myosin7a (Myo7a) (green) and enlarged views of the insets showing the stria vascularis of the apical, middle and basal cochlear canals. Of note, the brightness of the red channel was increased in overview images of the cohleae (top row) to distinguish tdTomato labelling both in the organ of Corti and in SGNs. Cell nuclei are stained in blue (DAPI).


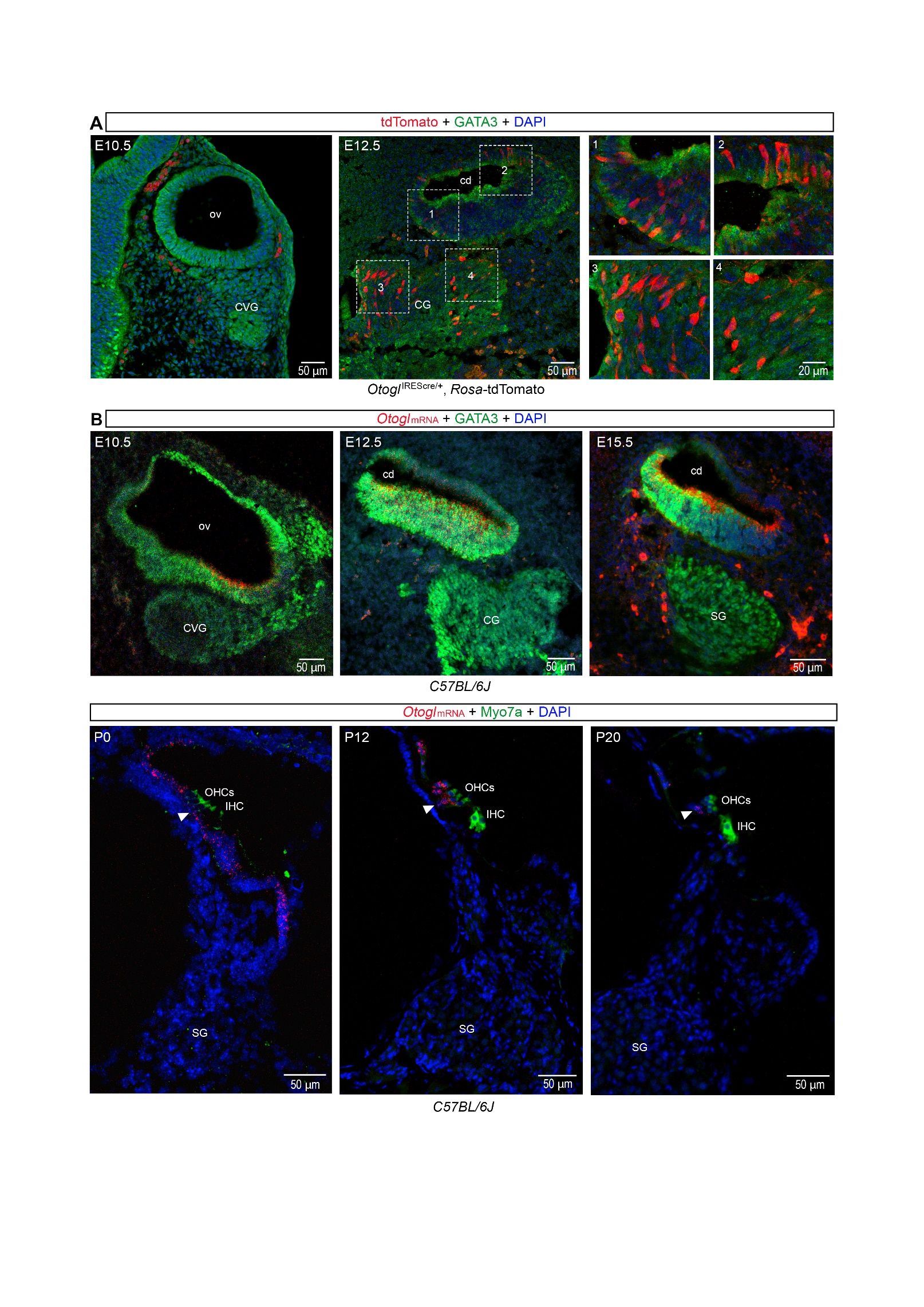


**Figure S8. Absence of *Otogl* mRNA in SGNs**

**A**) Longitudinal cross-sections of the inner ear of *Otogl*^IREScre/+^:*Rosa-*tdTomato mouse embryos on E10.5 (left) and E12.5 (middle and right), with immunostaining for GATA3 (green). Insets (right) show enlarged views of the cochlear duct and cochlear ganglion from the E12.5 mouse embryo. **B, top**) Cross-sections of the cochlear duct from wild-type mice on E10.5, E12.5, and E15.5, with the cochleo-vestibular ganglion (CVG) on E10.5, the cochlear ganglion (CG) on E12.5 and the spiral ganglion (SG) on E15.5 stained for *Otogl* mRNA and immunostained for GATA3. **B, bottom**) Cross-sections of the sensory epithelium and spiral ganglion from wild-type mice on P0, P12, and P20 stained for *Otogl* mRNA and immunostained for Myosin7a (Myo7a). Note that *Otogl* mRNA is absent from the spiral ganglion at all timepoints but detectable in the cochlear duct or the sensory epithelium. The arrows point to supporting cells containing *Otogl* mRNA used as an internal positive control. Cell nuclei are stained in blue (DAPI). cd, cochlear duct; CG, cochlear ganglion; CVG, cochleo-vestibular ganglion; IHC, inner hair cell; OHCs, outer hair cells; ov, otic vesicle; SG, spiral ganglion.


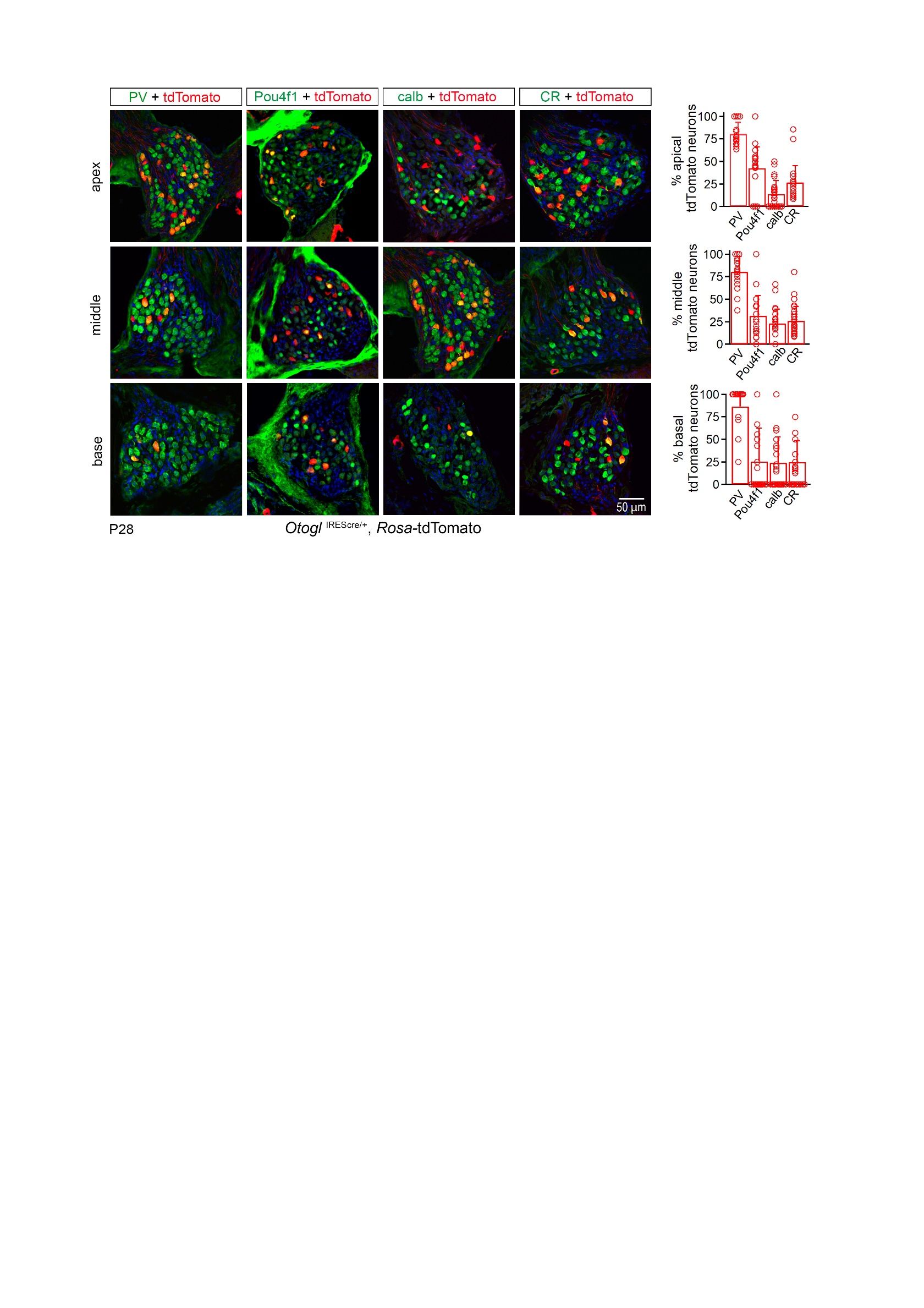


**Figure S9. TdTomato-positive neurons along the tonotopic axis of *Otogl*^IREScre/+^:*Rosa-*tdTomato mice**. Z-projections of the apical, middle and basal cochlear spiral ganglia cross-sections of a P28 *Otogl*^IREScre/+^:*Rosa-*tdTomato mouse immunostained for PV, Pou4f1, calb, or CR (left). Bar graphs show the proportion of tdTomato-positive neurons at the apex, middle, and base of the cochlea co-expressing PV, Pou4f1, calb, or CR (right). Cell nuclei are stained in blue (DAPI).


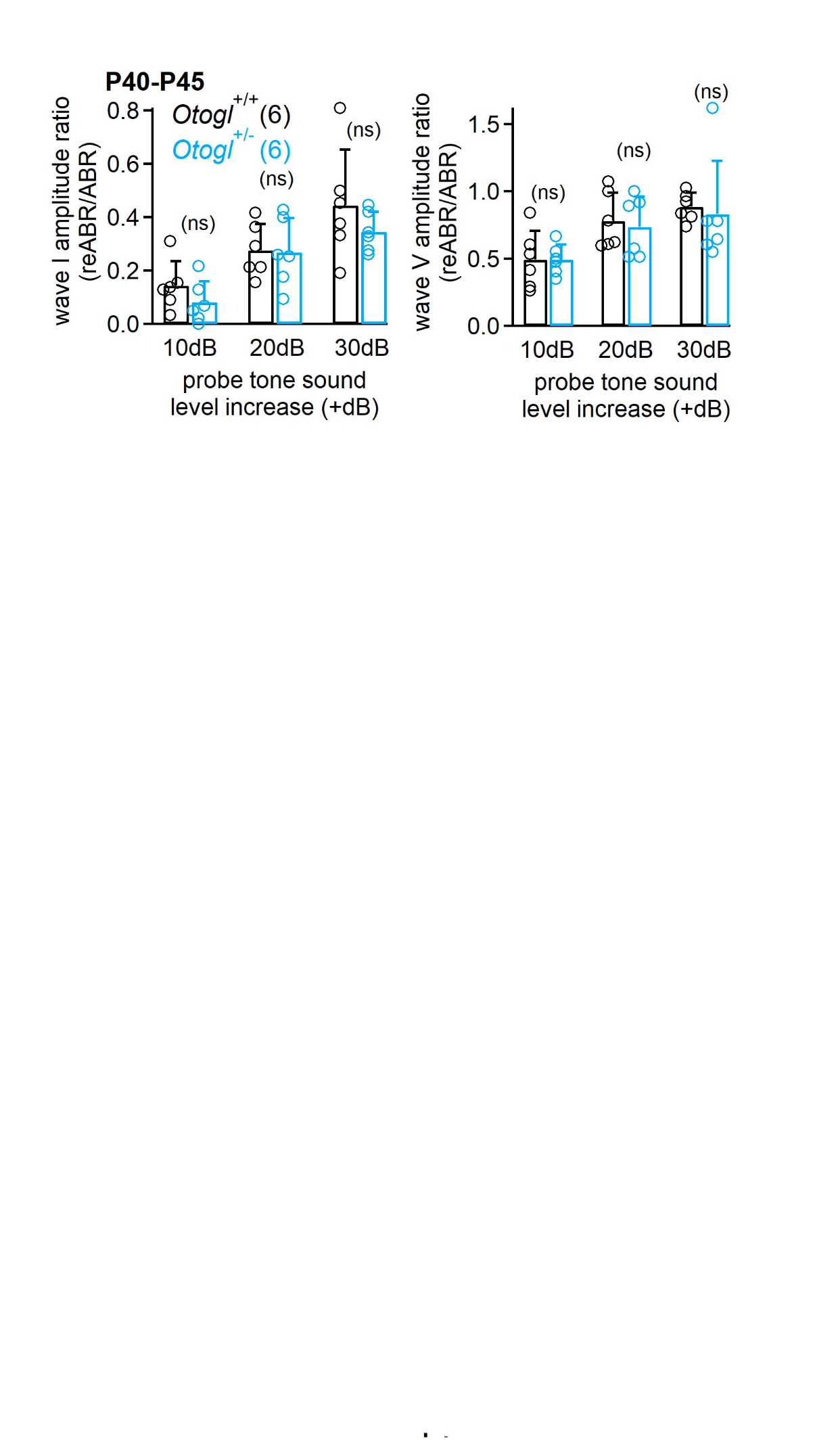


**Figure S10. Normal reABRs in *Otogl*^+/-^ P40-P45 mice**. Bar graphs showing the wave I amplitude ratio (reABR/ABR) (left) and the wave V amplitude ratio (right) in *Otogl*^+/+^ and *Otogl*^+/-^ mice on P40-P45 as a function of probe tone sound level increase. Numbers in brackets indicate the number of animals tested. ns, non-significant**.**


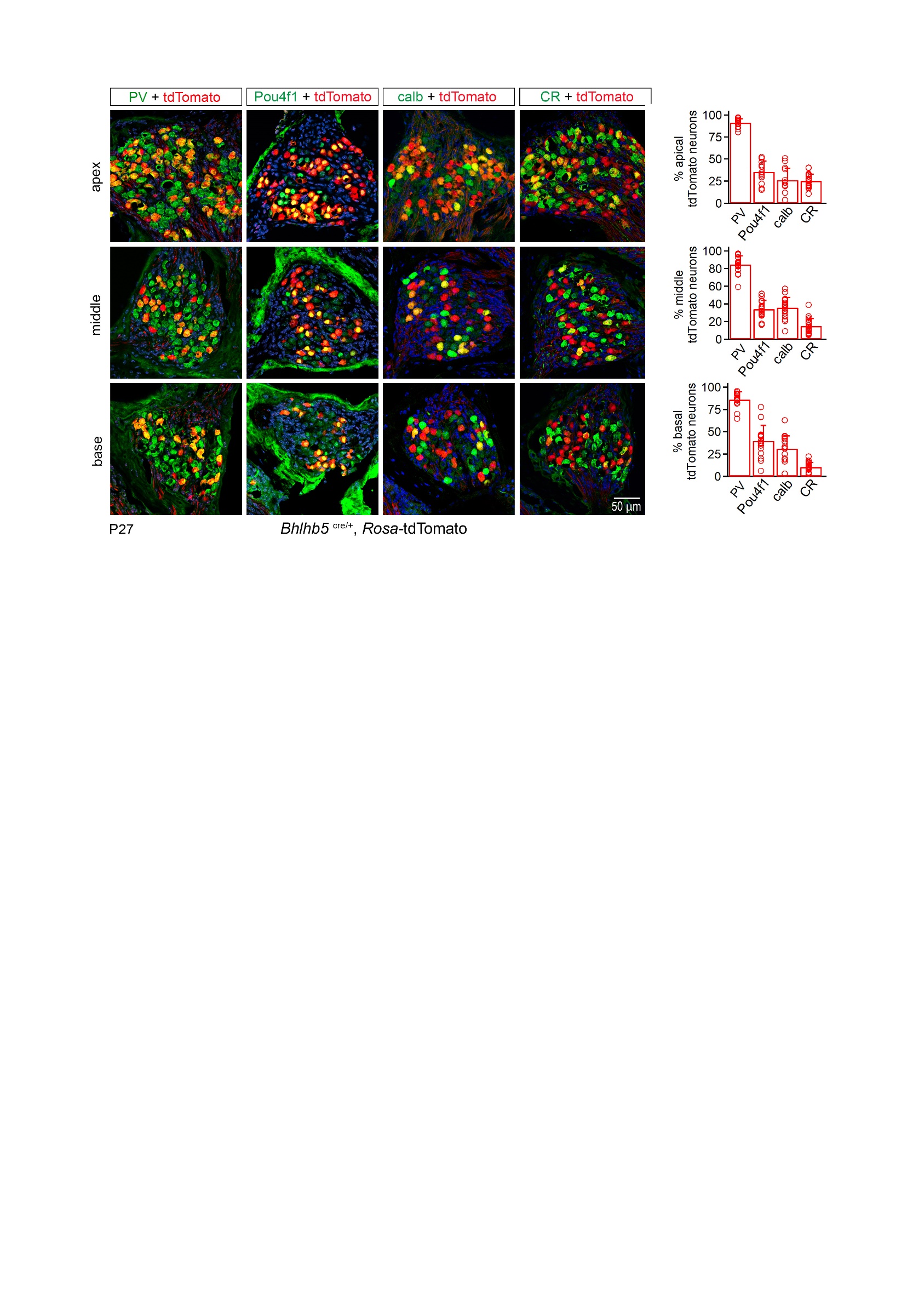


**Figure S11.** **TdTomato-positive neurons along the tonotopic axis of *Bhlhb5*^cre/+^:*Rosa-*tdTomato mice**. Z-projections of cross sections of apical, middle and basal cochlear spiral ganglia from a *Bhlhb5*^cre/+^:*Rosa-*tdTomato mouse on P28 immunostained for PV, Pou4f1, calb, or CR (left). Bar graphs show the proportion of tdTomato-positive neurons at the apex, middle, and base of the cochlea co-expressing PV, Pou4f1, calb, or CR (right). Cell nuclei are stained in blue (DAPI).
